# The RNA-binding protein hnRNP E1 regulates p53 and p21 translation *via* KH1 and KH2 domain interactions with 3′ UTR C-rich motifs

**DOI:** 10.1016/j.jbc.2025.111042

**Published:** 2025-12-12

**Authors:** Abhisekh Samanta, Arpita Kar, Sandipan Mukherjee, Avik Biswas

**Affiliations:** Department of Signal Transduction & Biogenic Amines, Chittaranjan National Cancer Institute, Kolkata, India

**Keywords:** hnRNP E1, KH domains, p53, p21, 3′UTR, protein-RNA interaction

## Abstract

Heterogeneous nuclear ribonucleoprotein E1 (hnRNP E1) is a member of the hnRNP family and contains three canonical K-homology (KH) domains. hnRNP E1 acts as a cancer antagonist by regulating specific transcripts, including the canonical oncogenic driver p53, as well as p21. This study aims to elucidate the molecular mechanisms underlying hnRNP E1–mediated regulation of p53 and p21, which remain largely unexplored. Here, RNA immunoprecipitation and photoaffinity crosslinking assays revealed that hnRNP E1 directly binds p53 and p21 mRNAs *via* specific C-rich RNA motifs in their 3′-UTRs, enhancing RNA stability and translation through increased polyribosome loading, as confirmed by polyribosome fractionation assays. Domain-deletion–based genetic mapping indicated that hnRNP E1 modulates both p53 and p21 expression primarily *via* its KH1 and KH2 domains. Photoaffinity crosslinking experiments further confirmed that KH1 and KH2 domains independently interact with the 3′-UTRs of both p53 and p21 mRNA, mimicking the functional activity of full-length hnRNP E1 in *in vitro* translation and cell-based luciferase reporter assays. Functional assays revealed that KH1 and KH2 domains of hnRNP E1 modulate p53 and p21 *via* UTR-guided mechanisms, leading to reduced proliferation, colony formation, and increased apoptosis. Upregulation of apoptotic markers (Bax, pro-caspase-8, caspase-3) was observed as a regulatory resultant of KH1 and KH2 domain-guided p53/p21 interaction. By establishing p53 and p21 as active components of the hnRNP E1 transcriptome, this study identifies KH domains as promising therapeutic candidates, warranting future investigation.

Dynamic alterations in the post-transcriptional regulation of various transcript species are a characteristic feature of numerous human diseases, including cancer (1, 2). Different post-transcriptional regulations are guided by various RNA-binding proteins (RBPs) (3). Heterogeneous nuclear ribonucleoproteins (hnRNPs) constitute an evolutionarily conserved family of RBPs (consisting of twenty major candidates, namely hnRNPs A1–U), which contribute to a wide range of cotranscriptional and post-transcriptional regulatory processes (4, 5). hnRNPs are RNA–protein complexes that act as key biomolecular machinery in cellular nucleic acid metabolism and homeostasis, also extending their modulatory functions to the proteomic level (6, 7).

While most hnRNP family members possess classical RNA recognition motifs, only two members, hnRNP E1 and hnRNP K, have canonical K-homology (KH) domains (8). Despite their high degree of structural similarity, hnRNP K promotes tumorigenesis by acting as an oncogene, whereas hnRNP E1 functions as a novel tumor suppressor and is downregulated in various cancer types (9, 10, 11, 12, 13, 14).

hnRNP E1 is widely expressed across diverse tissue or cell types, exhibiting both nuclear and cytoplasmic localization, and is encoded by an intron-less genetic element located on chromosome 2p12-p13 in humans (15, 16). All three KH domains of hnRNP E1 adopt a classical type I KH domain fold, characterized by a triple-stranded β-sheet packed against a three-helix cluster in the “β_1_α_1_α_2_β_2_β_3_α_3_” configuration (17). The three α-helices and three β-strands of the KH domains are arranged in an antiparallel configuration, facilitating a compact globular structure in which “β_1_α_1_α_2_β_2_” serves as the minimal KH domain capable of independently binding to the pyrimidine-rich RNA sequences with high affinity and specificity (17, 18, 19, 20). By binding with different transcripts, hnRNP E1 modulates their regulatory function and thereby governs their role in influencing cellular fate.

Previous studies have reported hnRNP E1–mediated proteome reprogramming *via* translational regulation during epithelial-to-mesenchymal transition, involving molecular events such as upregulation of p62 expression, stabilization of DKK1 mRNA, and subsequent suppression of β-catenin (21, 22, 23). Conversely, certain cellular factors, such as PARP1, exert a negative regulatory effect on hnRNP E1 (24). Some previous studies have characterized the molecular mechanisms of hnRNP E1 function in epithelial-to-mesenchymal transition, where hnRNP E1 was found to regulate the stability and translational control of different transcript species by targeting specific sequence motifs primarily located in the 3′-UTRs (25, 26, 27). However, considering the complex functional landscape of hnRNP E1, the intricate molecular mechanisms for the regulation of other facets of cancers particularly those involving critical cellular factors, by hnRNP E1 remain an open area to work on. Especially, how hnRNP E1 modulates key oncogenic drivers are still missing. Notably, previous studies have suggested a potential functional link between hnRNP E1 and gene expression profiles, including p53/p21 pathways (28, 29, 30). However, the functional mechanisms underlying hnRNP E1–mediated regulation of p53 and p21 are yet to be investigated. Thus, in the present study, the molecular basis of hnRNP E1–mediated regulation of p53, a key oncogenic driver (31), was investigated and the study further extended to depict the regulation of p21 too. Finally, through structure-to-function approach, the molecular basis of the function of hnRNP E1 (through specific KH domains) was documented to detail how specific hallmarks/features of cancers get modulated on the way to its anticancerous function.

## Results

### hnRNP E1 regulates p53 pathway by modulating p53 and p21 expression

**Figure 1:**
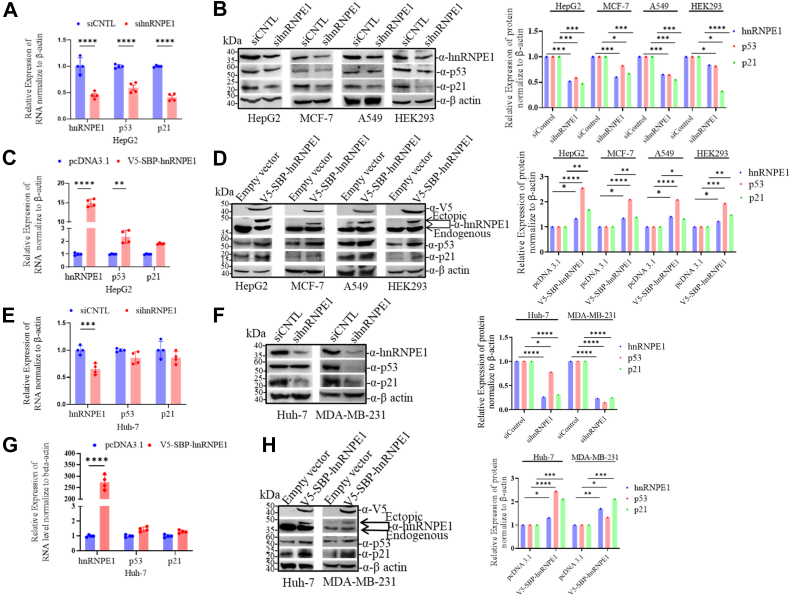
**hnRNP E1 silencing reduces p53 and p21 proteins but hnRNP E1 overexpression enhances p53 and p21 expression**. *A*, bar graph showing the effect of siRNA-mediated silencing of hnRNP E1 on p53 and p21 mRNA levels in HepG2 cells, quantified by real-time PCR (RT-PCR). *B*, Western blot panels showing the effect of hnRNP E1 silencing on p53 and p21 protein levels in HepG2, MCF-7, A-549, and HEK-293 cells. Bar graph (*right*) shows normalized blot band intensities for hnRNP E1, p53, and p21. *C*, bar graph showing the effect of hnRNP E1 overexpression on p53 and p21 mRNA levels in HepG2 cells using RT-PCR. *D*, Western blot panels showing the effect of hnRNP E1 overexpression on the p53 and p21 protein levels in HepG2, MCF-7, A-549, and HEK-293 cells. Bar graph (*right*) represents normalized blot band intensities of hnRNP E1, p53, and p21. *E*, bar graph showing the effect of hnRNP E1 silencing on p53 and p21 mRNA levels in HuH7 cells using RT-PCR. *F*, Western blot panels showing the effect of hnRNP E1 silencing on p53 and p21 protein levels in Huh7 and MDA-MB-231 cells. Bar graph (*right*) shows normalized hnRNP E1, p53, and p21 band intensities. *G*, bar graph showing the effect of hnRNP E1 overexpression on p53 and p21 mRNA levels in Huh7 cells using RT-PCR. *H*, Western blot panels depicting the effect of hnRNP E1 overexpression on the p53 and p21 protein levels in Huh7 and MDA-MB-231 cells. Bar graph (*right*) shows the normalized band intensities of hnRNP E1, p53, and p21. ∗*p* < 0.05, ∗∗*p* < 0.01, ∗∗∗*p* < 0.001, ∗∗∗∗*p* < 0.0001. Experiments in *A*, *C*, *E*, and *G* were replicated thrice, and those in *B*, *D*, *F*, and *H* were repeated twice across cell types. hnRNP, heterogeneous nuclear ribonucleoprotein.

Using *in vitro* cellular assays, we examined the regulatory effect of hnRNP E1 on p53 and p21 expression at both transcript and protein levels detected by using real-time PCR and Western blotting techniques. siRNA-mediated knockdown of hnRNP E1 in HepG2, MCF-7, A549, and HEK 293 cells (all are WT for p53) resulted in significant downregulation of p53 and p21 at both RNA and protein levels (Fig. 1, *A* and *B*, and S1, *A*, *C* and *E*). Conversely, ectopic overexpression of hnRNP E1 produced the opposite effect, leading to increased levels of both p53 and p21 transcripts and their respective translatants (Fig. 1, *C* and *D*, and S1, *B*, *D* and *F*). On repeating the same experiments in Huh7 and MDA-MB-231 cells (both harboring mutant for p53), comparable results were obtained (Fig. 1, *E*–*H*), indicating a direct effect of hnRNP E1 on p21 transcript/mRNA independent of p53.

Further experiments indicated that hnRNP E1 did not affect the promoter activity of either p53 or p21 (Fig. S2, *A* and *B*). In the p21 promoter assay, hnRNP E1 silencing led to the loss of luciferase signal in HepG2 and MCF-7 cells (expressing WT p53), whereas no such effect was observed in Huh7 and MDA-MB-231 cells (expressing mutant, transcriptionally inactive p53) (Fig. S2*B*). These observations clearly indicate that the effect on p21 promoter activity in HepG2 and MCF-7 cells was driven by hnRNP E1–mediated activation of WT p53 and further demonstrate that hnRNP E1 itself has no direct influence on p21 promoter activity.

### hnRNPE1 binds to the 3′-UTRs of both p53 and p21 enhancing transcript stability

**Figure 2:**
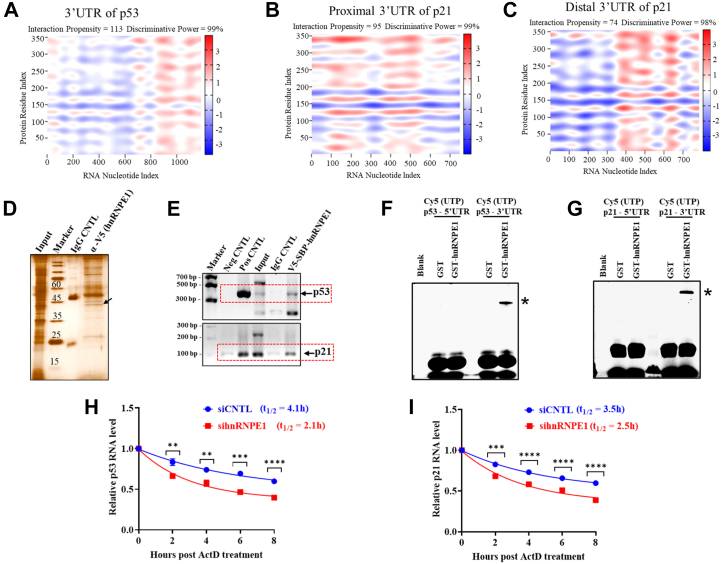
**hnRNP E1 interacts with 3′-UTRs of p53 and p21 RNAs and enhances their stability**. *A*–*C*, RNA–protein interaction heat maps generated using catRAPID, showing binding intensities of hnRNP E1 to the 3′-UTRs of p53 and p21; *x*-axis represents RNA nucleotide positions, and *y*-axis shows hnRNP E1 amino acid positions. Due to the large size of the p21 3′-UTR, the sequence was divided into proximal and distal segments for analysis. *D*, silver-stained SDS-PAGE gel showing the efficiency and specificity of RNA immunoprecipitation (RIP) using control (IgG) and V5 tag–based hnRNP E1 pulldown. *E*, agarose gel showing PCR-based confirmation of hnRNP E1 interactions with p53 and p21 RNAs in the RIP experiment. Dashed *red* boxes indicate the specific PCR-amplified products for p53 and p21. *F* and *G*, typhoon-scanned polyacrylamide gels from photoaffinity crosslinking experiment, showing direct interactions between 3′-UTRs of p53 and p21 with GST-hnRNP E1 fusion protein. Bands corresponding to the protein–RNA complexes are indicated with ∗. Lane 1 represents the blank control. No interaction was observed between the 5′-UTRs of either p53 or p21 with purified GST-hnRNP E1 fusion protein. *H* and *I*, mRNA decay assay for p53 and p21 under hnRNP E1-silenced conditions in HepG2 cells using actinomycin D, showing reduced mRNA stability upon hnRNP E1 depletion. ∗*p* < 0.05, ∗∗*p* < 0.01, ∗∗∗*p* < 0.001, ∗∗∗∗*p* < 0.0001. Data (*F*–*I*) originated from three independent experiments. hnRNP, heterogeneous nuclear ribonucleoprotein.

Since hnRNPE1 is an RBP and significantly influences the expression of both p53 and p21 mRNA (Fig. 1), we presumed that hnRNP E1 likely binds to both mRNAs. Using the catRAPID online protein–RNA interaction prediction tool (32, 33), we identified strong interaction propensities between hnRNP E1 and p53/p21 transcripts, primarily within their 3′-UTRs (Fig. 2, *A*–*C*, and S3), with positive interactions denoted with red in the heatmaps. Direct interactions between hnRNP E1 and p53/p21 transcripts were confirmed by RNA immunoprecipitation (RIP)-PCR assays (Fig. 2, *D* and *E*). Photoaffinity crosslinking experiments further showed that for both p53 and p21, the GST-hnRNP E1 fusion protein (Fig. S4*A*) strongly bound to both 3′-UTRs, while the 5′-UTRs showed no interaction (Fig. 2, *F* and *G*). Purified GST alone showed no interaction with any of the *in vitro* run-off UTR transcripts, thus ruling out any probable tag-specific effects.

To assess the effect of hnRNP E1 binding to the 3′-UTRs of both p53 and p21 mRNA, an actinomycin D–based RNA stability assays were performed under hnRNP E1–silenced conditions, revealing a significant decrease in the estimated stability of both RNAs (Fig. 2, *H* and *I*). Thus, it may be stated that hnRNP E1 upon binding to the 3′-UTRs of p53/p21 appears to increase their stability.

### hnRNPE1 targets specific C-rich RNA motifs within the 3′-UTRs of p53 and p21 mRNAs

**Figure 3:**
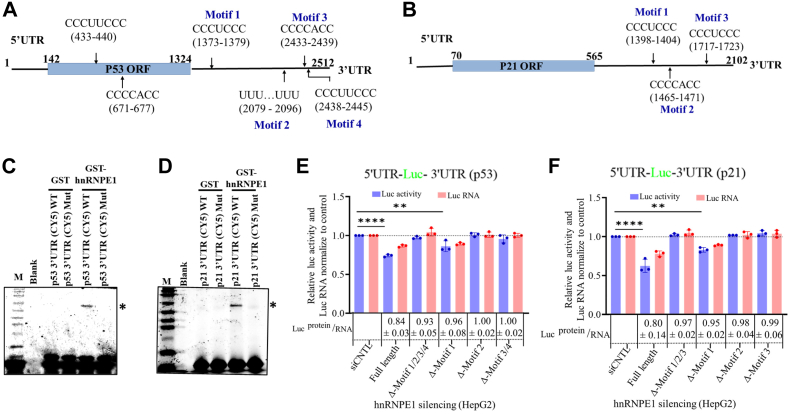
**hnRNP E1 binds to C-rich RNA motifs on 3′-UTRs of p53 and p21 and modulates their regulation**. *A* and *B*, predicted hnRNP E1 binding sites on p53 and p21 3′-UTRs were identified using catRAPID and are illustrated in schematic models of the respective mRNAs. *C* and *D*, typhoon-scanned polyacrylamide gels from photoaffinity crosslinking experiments show interactions between GST-hnRNP E1 fusion protein and WT 3′-UTRs of both p53 and p21. Bands corresponding to the interacting protein–RNA complexes are indicated with ∗. Motif-deleted mutant 3′-UTRs [3′-UTR(CY5) Mut; p53 Δ-motif 1/2/3/4 and p21 Δ-motif 1/2/3] showed no interaction (last lanes, both the gels). Lane 1 represents a blank control. No interaction of GST was observed with the 3′-UTRs of either p53 or p21. *E* and *F*, bar graph showing the functional role of each identified motif in the context of 3′-UTR–guided functional regulation of p53 and p21, by RT-PCR–based luciferase RNA levels and luciferase assays. HepG2 cell were transfected with either p53 or p21 5′-UTR-luciferase-3′-UTR expression plasmids or motif-deleted variants under hnRNP E1-silenced conditions (sihnRNP E1-mediated knockdown). Luciferase RNA levels and luciferase activity from siControl-transfected cells was normalized to 1, and relative luciferase RNA level and activity of WT and MUT (motif-deleted variants) 3′-UTRs is shown. The ratio of the normalized luciferase values to the normalized luciferase RNA levels are shown in the boxes below the bar graph. ∗∗*p* < 0.01, ∗∗∗∗*p* < 0.0001. Data represents findings from three independent experimental replicates. hnRNP, heterogeneous nuclear ribonucleoprotein.

We aimed to identify the specific RNA motifs within 3′-UTRs of p53 and p21 that are targeted by hnRNP E1. catRAPID analyses revealed no putative hnRNP E1–binding sites within the 5′ UTRs of either p53 or p21 (Fig. S3, *A* and *C*). Mentionable here, for p53, two sites popped out that are situated on the coding sequence/ORF (Fig. 3*A* and S3*A*) but were excluded from any further analysis, as when tested, both the p53 and p21 ORFs lacking native 5′- and 3′-UTRs showed no hnRNP E1–dependent changes in protein expression (Fig. S5). The RNA motifs located within the 3′-UTRs of both p53 and p21 exhibited a very high degree of sequence similarity, characterized by a specific pattern of conserved stretches, except for motif 2 of p53, which contained a unique poly U sequence (Fig. 3, *A* and *B*, and S3, *B* and *D*). Deletion of these identified RNA motifs (Fig. 3, *A* and *B*) from the UTRs of both p53 and p21 completely abolished hnRNPE1 binding (Fig. 3, *C* and *D*) as confirmed by photoaffinity crosslinking assays. Using a deletion-based functional motif screening approach with RT-PCR and luciferase-reporter assays, removal of RNA motif-1 in either p53 or p21 had minimal impact, as the luciferase transcript and related translational activity response did not return to baseline (Fig. 3, *E* and *F*). However, deletion of all identified motifs (*i*.*e*., Δ motif-1/2/3/4 for p53 and Δ motif-1/2/3 for p21), or individual deletion of motifs other than motif-1 (motif-2 or motif-3/4 for p53 and motif-2 or motif-3 for p21), resulted in the luciferase transcript and related translational activity (luciferase readouts) comparable to control levels under hnRNP E1–silenced conditions (Fig. 3, *E* and *F*), indicating their functional relevance. This luciferase assay readout patterns were consistent with the normalized luciferase RNA levels (Fig. 3, *E* and *F*), although the effect of hnRNP E1 silencing was somewhat more on luciferase activity response than the RNA levels, especially in case of full-length UTR and Δ motif-1 UTR for both p53 and p21.

### hnRNPE1 induces p53 and p21 mRNA translation in a 3′-UTR-dependent manner

**Figure 4:**
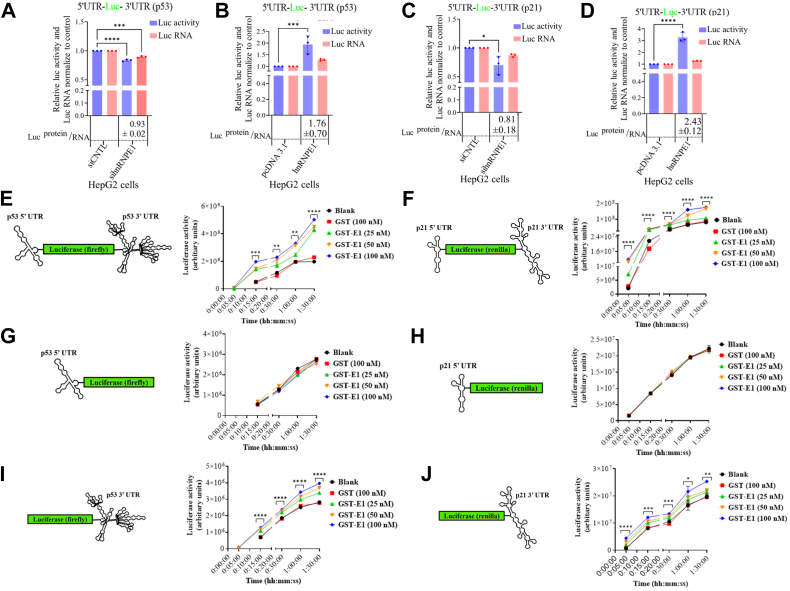
**hnRNP E1 induces translation of p53 and p21 mRNA *via* their 3′-UTRs**. *A* and *B*, bar graph showing the normalized (by setting control to 1) luciferase RNA levels using RT-PCR and luciferase assay–based translational efficiency under hnRNP E1-silenced or hnRNP E1-overexpressed cellular conditions in HepG2 cells transfected with p53 5′-UTR-luciferase-3′-UTR expression plasmid. *C* and *D*, bar graph showing the normalized (by setting control to 1) luciferase RNA levels using RT-PCR and luciferase assay–based translational efficiency under hnRNP E1-silenced or hnRNP E1-overexpressed cellular conditions in HepG2 cells transfected with p21 5′-UTR-luciferase-3′-UTR expression plasmid. Values in the boxes below the bar graph (A to D) indicate the ratio of normalized luciferase activity to luciferase RNA. *E* and *F*, line graph showing the *in vitro* translation assays of *in vitro* runoff transcripts containing both the 5′- and 3′-UTRs of p53 and p21 in the presence of increasing concentrations of purified GST-hnRNP E1 (GST-E1), showing time-dependent luciferase activity. *G* and *H*, similar to the experiment E and F, the *in vitro* translation assay for *in vitro* runoff transcripts containing only 5′-UTRs of p53 and p21. *I* and *J*, similar to the experiment E and F, the *in vitro* translation assay for *in vitro* runoff transcripts containing only 3′-UTRs of p53 and p21. All the experimental time points (5, 15, 30, 60, and 90 min) are presented in a linear scale. Schematic representations of transcript configurations (E to J) are provided. ∗*p* < 0.05, ∗∗*p* < 0.01, ∗∗∗*p* < 0.001, ∗∗∗∗*p* < 0.0001. Experiments were performed in triplicate. hnRNP, heterogeneous nuclear ribonucleoprotein.

We next examined the hnRNP E1–mediated, UTR-guided translation of p53 and p21 mRNAs by transfecting HepG2 cells with p53 or p21 5′-UTR-luciferase-3-’UTR expression plasmids under either hnRNP E1–silenced or hnRNP E1–overexpressed conditions. hnRNP E1 silencing (overexpression) led to decreased (increased) luciferase activity (Fig. 4, *A*–*D*), consistent with the corresponding normalized luciferase RNA levels. However, in hnRNP E1–silenced conditions, the p53/p21 UTR-guided decrease in luciferase readouts was more than the corresponding transcripts as the luciferase protein/RNA ratio <1 in all cases (Fig. 3, *E* and *F*; 4, *A* and *C*), while the exact opposite luciferase readout/RNA ratio (>1) was observed upon hnRNP E1 overexpression (Fig. 4, *B* and *D*). These results indicate that hnRNP E1 regulates p53 and p21 RNA abundance by modulating RNA stability and further links their translation rate in a UTR-dependent manner. hnRNP E1 did not affect the luciferase ORF (Fig. S6, *A* and *B*), thus ruling out any possible luciferase-driven effects.

During the *in vitro* translation assay, translation (in the form of luciferase activity) of p53 and p21 5-’UTR-luciferase-3-’UTR transcripts increased significantly over time subject to increasing concentrations of exogenously added GST-hnRNP E1 fusion protein (Fig. 4, *E* and *F*). Even at the lowest concentration tested (25 nM), the GST-hnRNP E1 fusion protein elicited a detectable translational signal within 5 min in the p53 5′-UTR-luciferase-3′-UTR assay (Fig. 4*E*). In contrast, when *in vitro* runoff transcripts lacking the 3′-UTRs, (5′UTR-luciferase only) were used, no changes in translational kinetics was observed, irrespective of the increasing concentration of GST-hnRNP E1 fusion protein (Fig. 4, *G* and *H*). However, when the *in vitro* runoff transcripts contained only the 3′-UTRs (luciferase-3′-UTR), hnRNP E1 significantly restored the translational induction for both p53 and p21 (Fig. 4, *I* and *J*). Notably, for p53, the 3′-UTR alone led to the detection of a translational signal at the 5-min time point only at the two highest hnRNP E1 concentrations (50 and 100 nM) (Fig. 4*I*). Collectively, these observations indicate that hnRNP E1 regulates the translation of p53 and p21 RNA through interaction with their 3′-UTRs.

**Figure 5:**
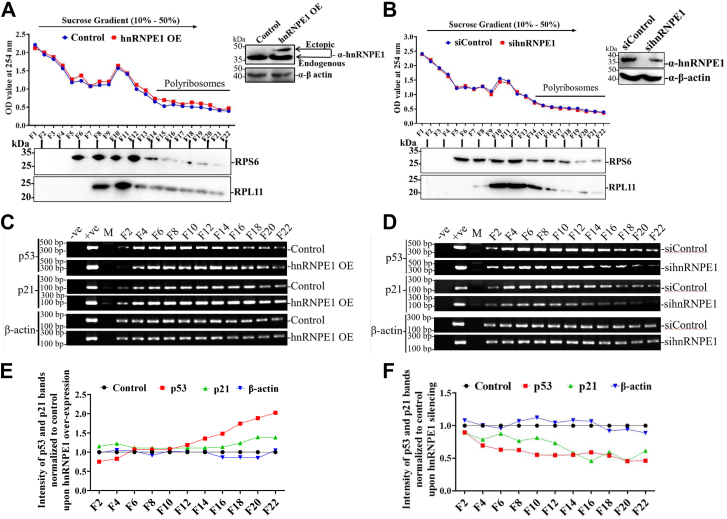
**hnRNP E1 induces p53 and p21 mRNA polyribosome loading**. *A* and *B*, ribosomal profiles from sucrose density gradient fractionation from HepG2 cells overexpressing or silenced for hnRNP E1. Fractionation efficiency demonstrated by probing for RPS6 and RPL11 distributions across alternative fractions with western blots. The hnRNP E1 silencing and overexpression are shown in the Western blot panels (*top right* of each subfigure). *C* and *D*, semiquantitative midphase PCR showing the relative abundance of p53, p21, and β-actin (control) mRNAs, in the collected alternative fractions from polyribosome fractionation assay following RNA extraction and cDNA synthesis. *E* and *F*, line graphs showing the relative band intensities of the p53, p21, and β-actin cDNA amplicons under hnRNP E1–overexpressed and hnRNP E1-silenced conditions relative to the control (normalized to 1). Data originated from three independent experiments. hnRNP, heterogeneous nuclear ribonucleoprotein. cDNA, complementary DNA.

Polyribosome fractionation assay under hnRNP E1–overexpressed conditions showed enhanced p53 and p21 mRNA loading onto polyribosomes (Fig. 5, *A*, *C* and *E*). Conversely, hnRNP E1 silencing produced the opposite effect (Fig. 5, *B*, *D* and *F*), as confirmed by semiquantitative PCR analysis of amplicon band intensities (Fig. 5, *C*–*F*). These results further indicate that hnRNP E1 plays a crucial role in p53/p21 polyribosome formation.

### KH1 and KH2 domains of hnRNP E1 regulates p53 and p21 expression

**Figure 6:**
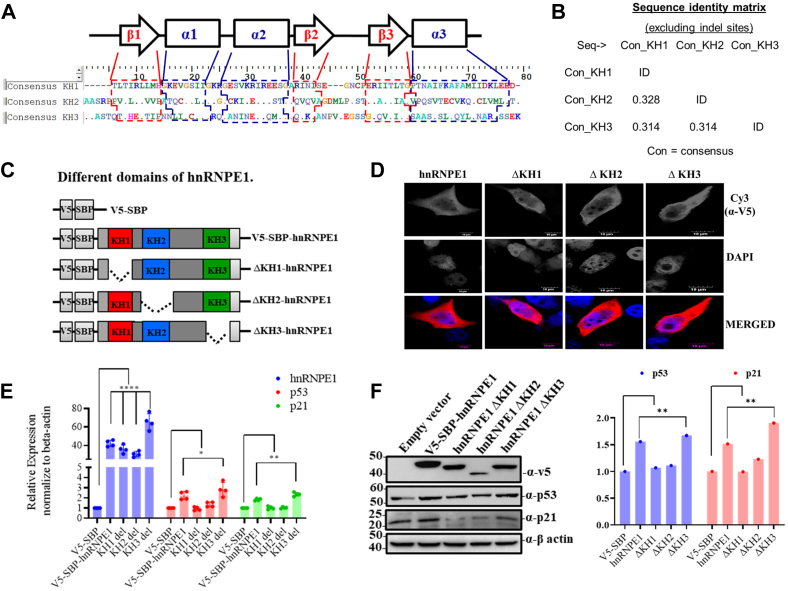
**KH1 and KH2 domains of hnRNP E1 mediates p53/p21 expression**. *A*, secondary structures of the KH domains of hnRNP E1 is shown. Dot matrix alignment of the three consensus KH domains (accession numbers NP_006187.2; CAA55016.1; NM_006196; BC039742 and X78137) is shown, where matches are shown as *dots* and the deletions/gaps as *dashes*. For positions of three α-helices (*blue*) and three β-sheets (*red*) of each KH domain are indicated with *dashed boxes* on the aligned sequences. *B*, sequence identity matrix showing the degree of sequence similarity among the three consensus KH domains of hnRNP E1. Matrix was generated using BioEdit software (version 7.2; https://bioedit.software.informer.com/7.2/#google_vignette) after excluding the indel sites. *C*, pictorial map depicting the location of three KH domains of hnRNP E1 and different KH-deleted variants of the hnRNP E1 expression system with V5 and SBP tags. *D*, intracellular localization of full-length hnRNP E1 and KH domain–deleted variants detected by immunofluorescence assay. The scale bar represents 10 μm. *E*, bar graph showing the relative expression of p53 and p21 RNA in cellular conditions overexpressing full-length hnRNP E1 or its different KH-deleted variants, measured by realtime PCR assay. *F*, Western blot analysis of p53 and p21 protein levels under overexpression of full-length hnRNP E1 or its different KH-deleted variants. Bar graph (*right*) shows normalized protein band intensities of p53 and p21. ∗*p* < 0.05, ∗∗*p* < 0.01, ∗∗∗∗*p* < 0.0001. The experiments (*D* to *F*) were replicated thrice. hnRNP, heterogeneous nuclear ribonucleoprotein.

Given that hnRNP E1 binds to specific RNA sequences primarily through its KH domains, we aimed to identify which domains are responsible for regulating p53 and p21 expression. Although the three KH domains of hnRNP E1 share similar secondary structures (Fig. 6*A*), they exhibit low amino acid sequence identity, approximately 32 to 33% (*i*.*e*., ≥ 67–68% variation) (Figs. 6*B* and S7*A*). Deletion of any of the KH domains from the full-length hnRNPE1 protein merely affects their gross cellular localization (Fig. 6, *C* and *D*). Notably, deletion of either KH1 or KH2 domain abolished hnRNP E1’s inductive effect on p53 and p21 expression at both the transcript and protein levels (Fig. 6, *E* and *F*).

**Figure 7:**
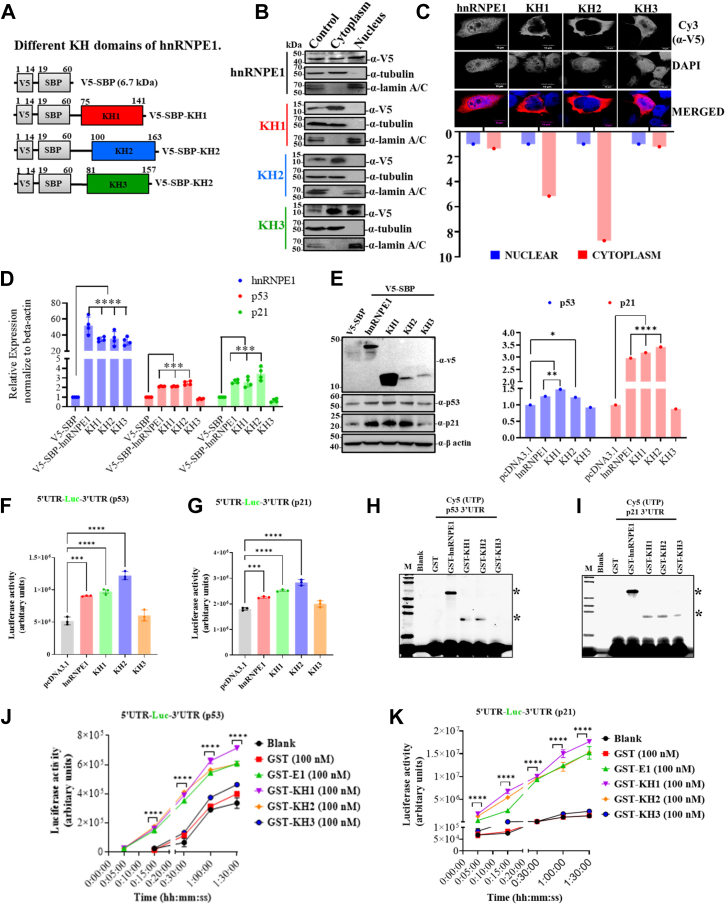
**KH1 and KH2 domains of hnRNP E1 independently binds to 3′-UTRs of p53/p21 and modulate their translation**. *A*, different KH domains with V5 and SBP tags are shown in the figure. *B*, Western blots showing nuclear versus cytoplasmic distribution of full-length hnRNP E1 and its KH domains based on fractionation assays. *C*, intracellular localization of hnRNP E1 and its KH domains detected by immunofluorescence assay. The scale bar represents 10 μm. Bar graph (*bottom*) shows the relative cytoplasmic localization scores normalized to nuclear localization score (set to 1). *D*, bar graph showing the relative expression of p53 and p21 RNA in cellular conditions overexpressing full-length hnRNP E1 or its different KH domains measured by real-time PCR. *E*, Western blot analysis of p53 and p21 protein levels under overexpression of full-length hnRNP E1 or its KH domains. Bar graph (*right*) displays the corresponding normalized protein band intensities. *F* and *G*, bar graph showing the effect of different KH domains on the translational efficiency measured using luciferase assays. HepG2 cells were cotransfected with each KH domain–encoding plasmids and either p53 5′-UTR-luciferase-3′-UTR (*F*) or with p21 5′-UTR-luciferase-3′-UTR (*G*) expression plasmids. *H* and *I*, typhoon-scanned polyacrylamide gels of the photoaffinity crosslinking experiments showing interactions of 3′-UTRs of p53 and p21 with GST-KH fusion proteins. Bands corresponding to the proteins–RNA complexes are indicated with ∗. Lane 1, blank control; GST-hnRNP E1 (full-length), served as positive control. *J* and *K*, line graph showing the time-dependent *in vitro* translation efficiency (luciferase signal) of *in vitro* runoff transcripts containing both 5′- and 3′-UTRs of p53 and p21, respectively, in the presence of purified GST-hnRNP E1 (GST-E1) and GST-KH (1/2/3) fusion proteins at equal concentrations (100 nM). ∗*p* < 0.05, ∗∗*p* < 0.01, ∗∗∗*p* < 0.001, ∗∗∗∗*p* < 0.0001. The experiments (*C*–*K*) were performed in triplicate. hnRNP, heterogeneous nuclear ribonucleoprotein.

When expressed individually in HepG2 cells (Fig. 7*A*), the KH3 domain showed a localization pattern similar to full-length hnRNP E1, present in both the cytoplasm and nucleus (Fig. 7, *B* and *C*). In contrast, the KH1 and KH2 domains were predominantly localized in the cytoplasm, as evidenced by both nuclear-cytoplasm fractionation and immunofluorescence assays (Fig. 7, *B* and *C*). The KH1 domain showed profound expression at the protein level, although no difference was noted at the RNA level (Fig. 7, *D* and *E*). Importantly, both KH1 and KH2 domains independently led to significant induction of p53/p21 expression at the level of mRNA and proteins (Fig. 7, *D* and *E*).

### KH1 and KH2 domains of hnRNPE1 independently interact with the 3′-UTRs of p53 and p21 to promote their translation

As deletion-based functional screening (Fig. 6) and KH domain–only expression assays (Fig. 7, *A*–*E*) indicated towards KH1- and KH2-mediated regulation of p53 and p21 translation, we next examined the specific functional mechanisms of each KH domain. Cotransfection experiments using either full-length hnRNP E1 or individual KH domain (1/2/3) constructs, along with p53 or p21 5′-UTR-luciferase-3′-UTR plasmids, resulted in a significant increase in the luciferase signal upon KH1 and KH2 overexpression (Fig. 7, *F* and *G*). These luciferase reporter assays confirmed that both KH1 and KH2 domains promote UTR-dependent translation of p53 and p21. However, the luciferase signal increased by approximately 1.5- to 2-fold (Fig. 7, *F* and *G*), which was lower than that observed in the case of KH1- or KH2-guided expression of endogenous p53/p21 (Fig. 7*E*), which might be due to different experimental platforms (endogenous p53/p21 expression versus transient luciferase reporter assay) and readout techniques.

Both GST-KH1 and GST-KH2 fusion proteins showed strong interactions with the 3′-UTRs of p53 and p21, whereas the GST-KH3 fusion protein showed a weak, tentative interaction with the p21 3′-UTR under the specified experimental settings (Fig. 7, *H* and *I*). The 5′-UTRs were excluded from these assays since they showed no interaction with GST-hnRNP E1 in previous experiments (Fig. 2, *F* and *G*). In *in vitro* translation assays, both GST-KH1 and GST-KH2 fusion proteins exhibited significant p53/p21 translational induction efficiency similar to or higher than that of the full-length hnRNP E1 protein (Fig. 7, *J* and *K*), demonstrating the independent and contextual functional capability of KH1 and KH2 domains.

### KH1 and KH2 domains can independently mediate diverse cellular regulatory phenotypes associated with tumorigenesis

**Figure 8:**
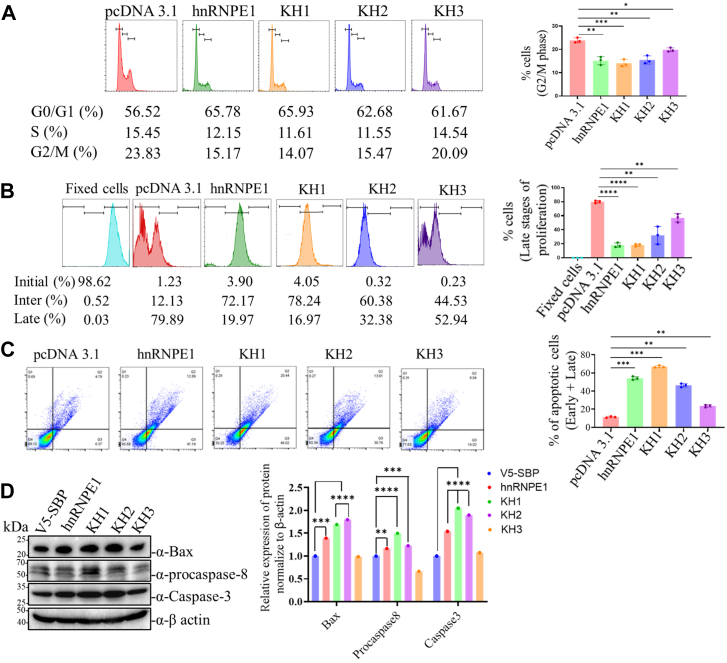
**Overexpression of KH1 and KH2 domains of hnRNP E1 induces anticancer-like phenotypes**. *A*, flow cytometry–based cell cycle assay depicting the percentages of cells in different stages of the cell cycle following the overexpression of full-length hnRNP E1 or different KH (1/2/3) domains in HepG2 cells. Average percentages for experimental triplicates are presented in a tabular format at the bottom of the FACS plots. Bar graph (*right*) depicts average percentages of cells in the G2/M phase. *B*, flow cytometry–based cellular proliferation assay showing the effects of overexpressing full-length hnRNP E1 or individual KH (1/2/3) domains on cellular propagation in HepG2 cells. Average percentages of cells present in three different experimental replicates are presented in a tabular format below the FACS plots; bar graph (*right*) shows the average percentages of cells in the late or advanced stage of cellular proliferation. *C*, flow cytometry–based cellular apoptosis assay showing the effect of overexpression of full-length hnRNP E1 or KH (1/2/3) domains on apoptosis in HepG2 cells. Bar graph (*right*) represents combined percentages of early and late apoptotic cells from three independent experiments. *D*, Western blot analysis of apoptosis-related markers, including Bax, procaspase-8, and caspase-3, at the protein levels when full-length hnRNP E1 or individual KH domains are overexpressed. Bar graph (*right*) represents the corresponding normalized protein band intensities. ∗*p* < 0.05, ∗∗*p* < 0.01, ∗∗∗*p* < 0.001, ∗∗∗∗*p* < 0.0001. hnRNP, heterogeneous nuclear ribonucleoprotein.

After assessing the mechanisms of KH1 and KH2 domain-mediated p53 and p21 modulation, the role of different KH domains was tested to correlate their regulatory effects on tumor-associated phenotypes. In cell cycle assays, both KH1 and KH2 domains alone significantly inhibited cellular transition to the G2/M phase (Fig. 8*A*), mirroring the effect of full-length hnRNP E1. Similarly, they suppressed cellular proliferation to a comparable extent (Fig. 8*B*). When tested, both KH1 and KH2 domains significantly induced apoptosis, at levels similar to full-length hnRNP E1 (Fig. 8*C*), correlating strongly with the expression of apoptosis-related markers, including Bax, procaspase-8, and caspase-3 (Fig. 8*D*). Additionally, the KH1 and KH2 domains inhibited colony formation (Fig. S8*A*). In scratch assay–based wound healing experiments, KH1 showed the strongest inhibitory effect, followed by KH2 (Fig. S8*B*). Collectively, these results support the independent anticancerous properties of the KH1 and KH2 domains of hnRNP E1.

## Discussion

The present study highlights the hnRNP E1–mediated molecular regulation of p53, a key canonical oncogenic driver (https://cancer.sanger.ac.uk/cosmic/census-page/TP53) that coordinately modulates different hallmarks of cancer (31). Furthermore, the study extends to its immediate downstream effector molecule p21, as both operate under a common functional axis. Some regulatory association studies suggested a potential functional link between hnRNP E1 and the p53/p21 pathway, with hnRNP E1 silencing or downregulation-related DNA damage response signal transduction *via* the p53 class and p21 as one of the differentially expressed candidates (28, 29). Our findings in the present study highlight the molecular regulation of the p53–p21 axis in the context of human cancers under the governance of the hnRNP E1 transcriptome.

Stretching the thread from the existing reports (34, 35), we confirmed the direct interaction between hnRNP E1 and p53/p21 mRNA using complementary approaches (Fig. 2, *D*–*G*). The hnRNP E1–mediated abundance in the RNA pool of both p53 and p21 across different cell types (Fig. 1, *A*, *C*, *E* and *G*) indicates that the observed molecular regulation is common across cancer types. The hnRNP E1–mediated regulation of p53 and p21 was driven by the interaction-based stability of the mRNAs rather than promoter activity–related transcriptional upregulation (Fig. 2, *H*, *I*, and S2). Regulation of mRNA stability or decay is a critical mechanism in controlling gene expression, which in turn depends on the interactions between cis-acting sequences generally present within the 3′-UTR and the trans-acting protein factors (RBPs) (35). Photoaffinity crosslinking experiments revealed that hnRNP E1 independently targets and binds to the 3′-UTRs of both p53 and p21 (Fig. 2, *F* and *G*). hnRNP E1 through its KH domains preferentially recognizes C-rich motifs localized to the 5′-CCCUCCC-3′ sequences within the UC-rich stretches (18, 36). Accordingly, catRAPID predictions were consistent with these experimental findings except for motif-2 of p53, which contains the poly-U stretch (Fig. 3). Deletion of these identified RNA motifs from the 3′-UTRs completely abolished the interaction with hnRNP E1 (Fig. 3, *C* and *D*). Notably, in the absence of these RNA-binding motifs (p53 3′-UTR-ΔM1/M2/M3/4 and p21 3′-UTR-ΔM1/M2/M3), the translational regulatory effect reflected in polyribosome formation disappears (Fig. S9, *A*–*D*, and S10, *A*–*D*), indicating that these C-rich RNA motifs are central to the functional regulation. Despite sharing a very high degree of sequence identity, including identical motifs (motif-1) (Fig. 3, *A* and *B*, S3, *B* and *D*), when located within or proximal to the coding sequence, fails to exhibit functional relevance (Fig. 3, *E* and *F*), probably due to some degree of ribosomal hindrance (37). Collectively, these observations strongly support the role of p21 3′-UTR as a composite cis-acting regulatory element (35), with the p53 3′-UTR exhibiting a similar regulatory profile.

KH domain–containing proteins are known to bind the 3′-UTRs of the actively translating RNA species and modulating both their half-life and post-transcriptional regulation (38, 39). Accordingly, hnRNP E1 increases the translation of both p53 and p21 transcripts in a concentration-dependent manner, indicating its vital and independent role in regulating their expression (Fig. 4, *E* and *F*). As expected, in the absence of 3′-UTRs (for both p53 and p21), which harbor the binding site for hnRNP E1, quite expectedly translational inductive effect is lost (Fig. 4, *G* and *H*). Conversely, when only the 3′-UTRs are present, the hnRNP E1–mediated translational induction is significantly restored (Fig. 4, *I* and *J*). However, the probable role of long-range 5′ and 3′-UTR interactions in p53 (40, 41) and p21 RNAs needs further study in this context. Beyond 5′-3′ UTR interactions, the p53 5′-UTR structure, featuring a large thermodynamically stable hairpin followed by a smaller stem-loop serves as a template for scanning by various translation factors, and in the absence of 5′-UTRs, such translation initiation–associated molecular affairs remain missing (42, 43). Notably, hnRNP E1 binding to a 3′-UTR of any given RNA species does not always necessarily lead to translational induction. Certain mRNAs, including those encoding for pro-oncogenic factors, can undergo translational silencing (25, 26, 27). A well characterized example is the 33-base-long RNA sequence [BAT element], which serves as a cognate binding site for hnRNP E1, and when located at the 3′-UTR of certain RNA molecules, represses their translation (25, 26, 27, 44). Consistent with previous reports, when tested, some of the pro-oncogenic factors, such as STAT3, EGFR, Jak2, *etc*. underwent induced expression at the level of their translatants upon hnRNP E1 silencing (data not shown). Thus, the translational regulation of any given RNA molecule by hnRNP E1 may well be determined by the nature and sequence of the specific RNA motif present within the 3′-UTRs.

The KH domains serve as the functional core of hnRNP E1, enabling its interaction with diverse RNA species, while differing in their RNA preferences (motifs) and regulatory outcomes (17, 20, 25, 36). Genetic and reverse-genetic approaches identified KH1 and KH2 as the key domains mediating p53/p21 regulation (Figs. 6 and 7). Notably, the KH1 and KH2 domains alone can effectively recapitulate full-length hnRNP E1 function in regulating the p53–p21 axis (Fig. 7, *D* and *E*), whereas the KH-deleted variants of hnRNP E1 (lacking either KH1 or KH2) fails to exert similar effects despite the presence of either KH1 or KH2 (Fig. 6, *E* and, *F*). Plausibly, the lack of analogous condition and their related orderliness in the absence of either KH1 or KH2 domains for domain-deleted hnRNP E1 variants might have some regulatory effect in the said context, as the function of any protein largely depends on the protein domain recurrence and their relative order (45, 46). Purified GST-fused KH1 and KH2 domains independently retain strong 3′-UTR-binding capacity and related translational regulatory activity on p53 and p21 *in vitro* runoff transcripts (Fig. 7, *H*–*K*), mirroring the functional mechanism of the full-length hnRNP E1. While only the KH3 domain harbors one nuclear localization signal ensuring its nuclear and cytoplasmic distribution, the perinuclear localization of KH1 and KH2 domains (Fig. 7, *B* and *C*) is primarily due to the absence of nuclear localization signal (Fig. S7*B*) and aligns with their translational regulatory functions, which occurs in the cytoplasm. The robust expression of the KH1 domain at the protein level (Fig. 7*E*) may be due to transcript-specific translational efficiency (47, 48) or RNA interaction–based gain of stability; however, needs future investigation. Collectively, these findings exemplify how the functional output arises from the complex interplay between primary amino acid sequences and the secondary structure of any within protein domains, such as the KH domains of hnRNP E1, a relationship that remains incompletely understood (49). Functional assay outcomes suggest that hnRNP E1 likely regulates a vast array of genes and molecular factors beyond p53 and p21, *via* KH1 and KH2 domains either directly or indirectly, in a largely intertwined manner, which requires future investigation. As KH1 and KH2 domain positively modulate the p53–p21 axis, a marked induction of apoptosis (Fig. 8, *C* and *D*), a major anticancerous event was observed. Small domains of some proteins can fully retain the affinity to interact with the putative cancer regulating cellular factors exerting regulatory functions (50, 51). Consistently, the KH1 and KH2 domains of hnRNP E1 independently regulates pivotal functional phenotypes, exerting anticancer activity across multiple facets including cellular proliferation, cell cycle, and colony formation, highlighting their potential to act as biologic against cancer.

In conclusion, this study catalogs p53 and p21 as active components of the hnRNP E1 transcriptome, establishing hnRNP E1 as a crucial regulator of their expression. The study elucidates the molecular intricacies of hnRNP E1–mediated translation of p53/p21 RNAs and demonstrates that individual KH1 and KH2 domains of hnRNP E1 are sufficient to regulate p53/p21 expression, with corresponding effects on anticancer phenotypes in humans. While hnRNP E1 has been speculated to hold therapeutic potential in anticancer treatment, (9, 10), our findings highlight that the KH1 and KH2 domains as central effectors, capable of selectively interfering with a plethora of RNA metabolic steps to exert context-specific anticancer activity.

## Experimental procedures

### Cell lines and plasmids

HepG2, MCF7, A549, HEK293, MDA-MB231, and Huh7 cells (STR profiled) were obtained from NCCS Cell Repository (https://www.nccs.res.in/cellrepository). Cells were grown and maintained in Dulbecco’s modified Eagle’s medium supplemented with 10% fetal bovine serum with or without the presence of antibiotic (100 U/ml penicillin and 100 μg/ml streptomycin) as needed in 5% CO_2_ containing humidified chamber. Nonessential amino acids (1X final concentration) were added as required. Additional G418 at a working concentration of 1 μg/ml was added to the media to select the transformants.

For the overexpression of hnRNP E1, plasmid pT7-V5-SBP-C1-HshnRNP E1 was used which was a gift from Elisa Izaurralde (Addgene plasmid # 64921; http://n2t.net/addgene: 64921; RRID: Addgene_64921) and upon transfection, resulted in robust hnRNPE1 expression in the RNA levels (viz Fig. 1*C*, 1*G*, 6*E*, 7*D*, S1*B*, *D*, *F*, etc) ultimately leading to modest ectopic expression at the protein levels (viz Fig. 1, *D* and *H*, etc). For WT p53 overexpression, plasmid pcDNA3 p53 WT was used which was a gift from David Meek (Addgene plasmid # 69003; http://n2t.net/addgene: 69003; RRID: Addgene_69003). For the p21 WT expression system, the p21 ORF sequence was cloned in pcDNA 3.1 (+) backbone with C-term His-tag at the BamH1 site. For the p53 promoter assay, plasmid pGL2-356 bp was used, which was a gift from Wafik El-Deiry (Addgene plasmid # 16292; http://n2t.net/addgene:16292; RRID: Addgene_16292). For the p21 promoter assay, pGL2-p21 promoter-Luc was used which was a gift from Martin Walsh (Addgene plasmid # 33021; http://n2t.net/addgene: 33021; RRID: Addgene_33021). For p53 5′-UTR-luciferase-3′-UTR expression cassette, 145-pGL3ctrl-3′ plasmid was used which was a gift from Michael Kastan (Addgene plasmid # 28175; http://n2t.net/addgene: 28175; RRID: Addgene_28175). For the p21 5-’UTR-luciferase-3′-UTR expression cassette, in the pGL4.74 plasmid, the p21 5′-UTR sequence was cloned at HindIII site immediately upstream of the luciferase (hRLuc) sequence and the p21 3′-UTR sequence was cloned at XbaI site immediately following the stop codon of the luciferase (hRLuc) sequence (Fig. S11). For all the in-house cloning purposes, complementary DNA (cDNA) prepared from total RNA extracted from HepG2 cells was used to generate the inserts.

For the generation of the deletion mutants from pT7-V5-SBP-C1-HshnRNP E1 (either KH domain–deleted versions of hnRNP E1 or individual KH domain only versions *i*.*e*. V5-SBP-KH-1/2/3 only), a PCR-based restriction-free cloning approach was employed (52, 53). For expression and purification of GST-fused hnRNP E1 or the GST-fused three KH-only domains, clones were prepared with pGEX-6p-1 backbone at EcoRI site.

All the cloned genetic fragments were checked with PCR and confirmed with sequencing. The details of the primers used in the study are presented in Table S1.

### siRNA and plasmid DNA transfections

For silencing the endogenous levels of target proteins, for every 10^5^ cells [for HepG2, MCF7, A549, HEK293, MDA-MB231, Huh7], 1.0 μl of pooled siRNA duplexes (10 μM concentration) were transfected with reverse transfection using Lipofectamine RNAiMAX Transfection Reagent (Thermo Fisher Scientific). The siRNA duplexes used in the study, the pooled irrelevant siRNA control (scramble or irrelevant control, sicontrol; 4404021) and siRNA hnRNP E1 (AM16708; ID 144217), are from Ambion. The sequence of the siRNA used for knocking down the expression of endogenous hnRNP E1 is 5′-GGCGGGUGUAAGAUCAAAGtt-3′. The hnRNP E1 knockdown efficiency of used siRNA is shown in Figure 1, *A*, *B*, *E*, and *F*.

For transfection of plasmid DNA, Lipofectamine 2000 was used as per the manufacturer’s instructions.

### RNA immunoprecipitation

The protein RNA co-IP experiment was done as described previously with minor modifications (54). Briefly, HepG2 cells were transfected with pT7-V5-SBP-C1-HshnRNP E1 plasmids and 48 h post-transfection, cells were lysed with RIP lysis buffer [150 mM KCl, 25 mM Tris–HCl (pH 7.4), 5 mM EDTA, 1% Triton X-100, 2.5 mM DTT, 1X Complete protease inhibitor (Roche), and 100 U/ml Rnase inhibitor (RNasin Ribonuclease Inhibitor, Promega)]. After centrifugation at 16,000 x g for 30 min at 4 °C, the supernatant was precleared with uncoupled IgG beads with gentle shaking at 4 °C, and after preclearing, the supernatant (500 μl each) was incubated with either IgG coupled (to serve as control) or anti-V5 antibody–coupled A/G magnetic beads (PureProteome Protein A/G Magnetic Beads, Millipore) and incubated at 4 °C for 4 h with gentle rotation with uniform mixing. After incubation, the beads were separated from the supernatant and washed three times with wash buffer [150 mM NaCl, 50 mM Tris–HCl (pH 7.5), 1 mM EDTA, 0.5% NP-40, 10% glycerol, and 100 U/ml Rnase inhibitor]. The washed beads were directly used for RNA extraction using QIAGEN RNeasy Mini Kit (Cat. no: 74104), and subsequently, cDNA was synthesized from the extracted RNA with EvoScript Universal cDNA Master (Roche; Cat. No: 07912374001) using random hexamer primers.

### Quantitative real-time PCR

Briefly, following RNA extraction from the cultured cells using QIAGEN RNeasy Mini Kit (Qiagen, Cat no. 74104), 1 μg of RNA was reverse transcribed with EvoScript Universal cDNA Master (Roche) using random hexamer primer. The prepared cDNA was diluted five times with nuclease-free water and 1 μl of diluted cDNA was used for each reaction of real-time PCR using FastStart Essential DNA Green Master (Roche) in LightCycler 96 Instrument. The relative expression of the target genes was calculated with 2^–ΔΔCt^ method.

### RNA stability assay with actinomycin-D

HepG2 cells were transfected with siRNAs (siControl and sihnRNP E1) and 48 h post-transfection, actinomycin-D (final concentration 5 μg/ml) was added to each of the transfected cells. At each time point (0, 2, 4, 6, and 8 h), RNA was extracted using QIAGEN RNeasy Mini Kit from the transfected cells (both for siControl and sihnRNP E1) and cDNA was prepared. The relative expression for p53 and p21 was measured with real-time PCR as described above and the decay calculation was done with GraphPad Prism version 9.3.1.

### Western blotting

Following cell lysis with lysis buffer (50 mM Tris–HCl (pH 7.4), 150 mM NaCl, 1 mM EDTA, and 1% Triton X-100) containing protease inhibitor cocktail, 10 μg of each protein lysate was resolved in SDS-PAGE gel and electroblotted onto nitrocellulose membrane. The membrane was blocked with PBS or TBS + 0.1%Tween 20 containing 5% nonfat dried milk for 2 h at room temperature followed by overnight incubation with primary antibodies. The next day, the membrane was washed three times with PBS or TBS + 0.1%Tween 20 and incubated with the Horseradish peroxidase-conjugated secondary antibody at room temperature for 1 h. After that, the membrane was washed 3X with PBS or TBS + 0.1% Tween 20 and blots were developed using either SuperSignal West Pico PLUS Chemiluminescent Substrate or with SuperSignal West Femto Maximum Sensitivity Substrate (Thermo Fisher Scientific).

The details of the antibodies used in the study are as follows: anti-V5-tag antibody (D3H8Q) from CST; anti-hnRNPE1 antibody (A1044) ABclonal; p53 (9282) CST; p21 (2947) CST; beta-actin (MA5-15739) Invitrogen; beta-tubulin (MA5-16308) Invitrogen; Lamin A/C (2032) CST; bax (2772) CST; caspase-8 (4790) CST, and caspase-3 (9662) CST.

The specificity and size of the detected/probed proteins using Western blotting methods are confirmed by comparing with the respective bands of the protein ladder (Figs. 1, 5–8). For the quantification of the protein band intensities of western blots, the protein band panels were analyzed with the ImageJ software (https://imagej.net/ij/) to assess/estimate the individual protein band intensities (in numerical values). After quantification of the target protein bands (*viz*. hnRNP E1, p53, p21, bax, procaspase-8, and caspase-3), their relative expression was normalized with the quantified protein band intensities of the respective controls (β-actin) and the degree of expression of the target proteins are depicted with bar graphs (Figure 1, Figure 6, Figure 7, Figure 8). Additionally, for hnRNPE1 silencing and overexpression experiments (Fig. 1), the relative expression of hnRNP E1, p53, and p21 were normalized to 1 for control siRNA and control plasmid transfected cells respectively and subsequently the normalized relative expression of hnRNP E1, p53, and p21 in hnRNP E1–silenced or overexpressed cells are shown in bar graphs across the different types of cell lines.

### Expression and purification of GST fusion proteins

The hnRNP E1 and the individual KH domains were cloned into the pGEX6p1 plasmid. The GST-hnRNP E1 and GST-KH (KH1, KH2, and KH3) fusion proteins are expressed in *Escherichia coli* BL21 (DE3) cells and purified with Glutathione Sepharose Beads (GE Healthcare). Briefly, 1 L LB broth cultures of BL21 cells transformed with the GST, GST-hnRNP E1, and GST-KH (KH1, KH2, and KH3) fusion proteins cloned expression vector were grown at 37 °C until an A600 of 0.6 was reached. Cells were then induced by adding 1 mM IPTG and grown for 4 h at 37 °C with vigorous shaking. Cells were then harvested at 5000 x g for 10 min, washed with 1X PBS, and resuspended in 50 ml of cold lysis buffer A (25 mM Tris–HCl pH 7.8, 250 mM NaCl, 0.5 M EDTA, and 1X Complete protease inhibitor) and lysed by sonication (5 pulses for 15 continuous seconds with 1 min pause in between using amplitude settings of 7 out of 10) with the sonicator (Hielscher; UP200S). Mentionable here, during the lysis of the GST-KH1 and GST-KH2 expressing BL21 (DE3) cells, sarkosyl (0.1% final concentration) was added to the lysis buffer to increase the amount of GST-KH1 and GST-KH2 fusion proteins in the soluble fraction. Lysates were clarified by centrifugation at 160,00 × *g* for 20 min at 4 °C and further passage through a 0.45 μM filter. Clarified lysates were mixed with pre-equilibrated GST beads slurry and incubated at 4 °C for 3 h with gentle shaking. Following binding, bead slurry was washed extensively with binding buffer A, and elution was performed with binding buffer A supplemented with 10 mM reduced glutathione. For the eluted fraction of the proteins, overnight dialysis was performed with 1X PBS, pH 7.4 (AccuGENE PBS, Lonza) to remove glutathione and protein concentration was done with 10 kDa centrifugal concentrator (Amicon Ultra, Millipore), aliquoted, and stored at −80 °C until further used. The purity of proteins was assessed by SDS-PAGE and concentrations were determined by BCA assay (Pierce).

### T7 polymerase-based *in vitro* runoff transcription

For the preparation of *in vitro* transcripts, mMESSAGE mMACHINE T7 Transcription Kit (AM1344; Invitrogen) was used as per the manufacturer’s instruction. Briefly, for the amplification of linear templates, forward primers with 5′ T7 promoter overhangs were used (Table S1). In a 20.0 μl reaction mixture, 500 ng of purified linear DNA template was used and the reaction was performed at 37 °C for 2 h. After a 15-min digestion with Turbo DNase, the yielded RNA was extracted using QIAGEN RNeasy Mini Kit, measured, and stored at −80 °C for further use.

### Photoaffinity crosslinking assay

Purified GST or GST-hnRNP E1 or GST-KH (KH1, KH2, and KH3) fusion proteins (500 ng) were photochemically crosslinked with p53 and p21 Cy5-labeled 5′-UTR or 3′-UTR transcripts (100 ng). Briefly, the incubation buffer contained 50 mM Tris–HCl (pH 7.9), 1.5 mM DTT, 1 mM MgCl2, and 0.01% bovine serum albumin in a final volume of 25 μl. First, the mix was incubated on ice for 30 min, followed by UV irradiation at 300 mJ/cm2 (GS GENE Linker, Bio-Rad). The irradiated samples were treated with RNase A (0.1 μg/μl; Cayman Chemical) for 15 min at 37 °C and resolved the crosslinked RNA–protein complexes on 10% SDS polyacrylamide gel. The labeled RNA–protein complex was visualized using a Typhoon scanner (Fluorescent Image Analyzer, Amersham Typhoon, GE). The scanned gels were stained with Coomassie blue to confirm the location of the protein–RNA complexes.

### Luciferase assay

For luciferase assay (either firefly or renilla luciferase assay), Promega’s luciferase assay kit was used as per the manufacturer’s instructions (E1500 and E2810, respectively). The luciferase readings were recorded using a luminometer (Promega, GloMax 20/20).

### p53 & p21 UTR assays

HepG2 cells were reverse transfected with siRNAs (siControl and sihnRNP E1) as described earlier in a 12-well plate and, after 12 to 16 h, the cells were directly transfected with p53 5′UTR-luciferase-3′UTR expression plasmid (145-pGL3ctrl-3′ plasmid) and/or pGL4.74 p21 5′UTR-luciferase-3′UTR plasmid in different sets of experiments. There were three replicates for each experiment. After 48 h of DNA transfection, cells were lysed and a luciferase assay was done.

### *In vitro* translation assay

*In vitro* translation of the *in vitro*–transcribed runoff transcripts (50 ng each per reaction) were carried out in micrococcal nuclease-treated rabbit reticulocyte lysates (RRL, Promega Corp.) with differing concentrations of purified GST-hnRNP E1 or GST-KH (KH1, KH2, and KH3) fusion proteins while blank (no protein control) and GST were used as controls. Briefly, 80 μl reaction mixtures containing 50 μl of RRL (62.5%), 1.0 μl each of Met^-^ (minus) and Leu^-^ (minus) amino acid mixtures, and 100 units of RNasin (RNasin Ribonuclease Inhibitor, Promega) was prepared. The reaction mixtures were incubated at 30 °C and allowed to continue for 90 min representing five different experimental time points (5, 15, 30, 60, and 90 min). At every time point, 9.0 μl of reaction mix was removed and mixed with 200 μl of ice-cold 1x luciferase lysis buffer containing cycloheximide (CHX, 100 μg/ml) and stored on ice to ensure no further activity. From each time point, mixture of 20 μl was used for three independent luciferase assays/measurements and the raw values are directly plotted in the graph (*y*-axis). The experimental conditions, *that is*, concentrations of RRL, concentrations of the *in vitro* runoff transcripts (50 ng per reaction), assay duration for the reaction kinetics (time on a linear scale; plotted on the *x*-axis) etc were kept constant. During the experiments, purified GST-only and no protein (blank) served as controls. Mentionable here, the purified GST-only showed no effect on the translational kinetics over time as compared with the blank control in any of the conducted *in vitro* translation assay experiments.

### Polyribosome fractionation assay

Analysis of p53/p21 RNA loading on polyribosomes was done as described previously with minor modifications (55, 56, 57). Briefly, HepG2 cells in 60 cm^2^ plates were transfected with the hnRNP E1 overexpression and null plasmids (to serve as control). Seventy-two hours post-transfection, cells were lysed and used for polyribosome fractionation analysis. Briefly, cells were treated with cycloheximide (CHX, 100 μg/ml) for 5 to 10 min, washed once in 1X PBS (containing 100 μg/ml CHX), and then lysed in polysome lysis buffer [10 mM Hepes (pH 7.4), 5 mM MgCl2, 100 mM KCl, 0.5% NP-40, 100 μg/ml CHX, and 1X Complete protease inhibitor (Roche) and 100 U/ml Rnase inhibitor (RNasin Ribonuclease Inhibitor, Promega)]. Cell lysates were passed five times with a 26G needle, spun at 12000 x g (5 min), the supernatant was loaded onto a linear density (10%-50% W/V) sucrose gradient [10 mM Hepes, 5 mM MgCl2, 100 mM KCl, 100 μg/ml CHX, 1X Complete protease inhibitor (Roche), and 100 U/ml Rnase inhibitor (Promega)], and subjected to ultracentrifugation (36,000 rpm for 2 h, at 4 °C) in a Beckman SW41 rotor (Beckman Coulter, Model: Optima XPN-100). From top to bottom, 22 fractions of 500 μl were collected and the OD of each fraction was measured at 254 nm with a spectrophotometer (Evolution 350, Thermo Fisher Scientific) as described previously (58). Similar experiments were performed with siRNA-mediated hnRNP E1–silenced condition too.

Based on the OD values, from 11 alternative collected fractions (out of 22), 250 μl was used directly for RNA extraction (RNeasy Mini Kit, Qiagen) and the rest was used for Western blotting experiments. Out of 25 μl eluted RNA, 12.0 μl used for cDNA synthesis in 20 μl reaction volume with random hexamer primers (EvoScript Universal cDNA Master, Roche). Finally, the synthesized cDNA was diluted twice with 20 μl nuclease-free water. The distributions of both p53 and p21 RNA among the different fractions were assessed with semiquantitative PCR by assessing the band intensity of the PCR-amplified products. The fractionation efficiency and the monosome fractions were confirmed by probing the collected fractions for RPS6 and RPL11 with immune blotting.

### Confocal immunofluorescence assay

Briefly, HepG2 cells were plated in a four-well chamber slide. After transient transfection with pT7-V5-SBP-C1-HshnRNP E1 plasmid and its KH domain–deleted variants or V5-SBP-KH-1/2/3–only variants of overexpression plasmids, cells were stained for V5 tag (where nontransfected cells served as negative control confirming the specificity of the used anti-V5 tag-specific antibody). In all cases, 1:500 dilution was used for both primary and secondary antibodies. All the images were captured under a confocal fluorescence microscope (Olympus FLUOVIEW FV4000 with DP 75 camara) at 100X magnification. The captured images were processed (visualization, color imposition, and merging of channels) using ImageJ software (https://imagej.net/ij/). The same software was used for the analysis of the distribution and quantification of particular proteins in the nucleus and cytoplasm following steps as mentioned elsewhere (59).

### Cell cycle assay

For cell cycle assay, HepG2 cells were either reverse transfected with siRNAs (for control CNTL siRNA and for experiment hnRNP E1 siRNA) or transfected with different plasmids (null plasmid, pT7-V5-SBP-C1-HshnRNP E1 and V5-SBP-KH-1/2/3 only variants). Post 24 h of transfection, cells were synchronized with nocodazole (50 ng/ml) for 8 h. After 36 to 48 h of synchronization, the transfected cells were detached from the plate and fixed and kept at −20 °C for 2 h. After fixation, cells were treated with RNaseA and stained with PE (Cayman, ID: 10009349), then incubated for 20 min in the dark then analyzed with flow cytometry (BD LSRFortesa X-20, model no-657666R1).

### Cells proliferation assay

HepG2 cells were stained with CFSE dye (Cayman, ID: 10009853) according to the manufacturer’s protocol and reverse transfected with siRNAs (for control CNTL siRNA and for experiment hnRNP E1 siRNA) or directly transfected with different plasmids (null plasmid, pT7-V5-SBP-C1-HshnRNP E1 and V5-SBP-KH-1/2/3–only versions). Poststaining, the leftover cells were fixed with 2% paraformaldehyde and stored at 4 °C (as initial). Post 24 h of transfection, cells were synchronized with nocodazole (50 ng/ml) for 8 h. After 48 h of transfection, cells were detached from the plate and analyzed with flow cytometry.

### Apoptosis assay

HepG2 cells were seeded in a 6-well plate; post 14 to 16 h of seeding, cells were directly transfected with different plasmids (null plasmid, pT7-V5-SBP-C1-HshnRNP E1, and V5-SBP-KH-1/2/3–only versions). Post 48 h of transfection, cells were stained with annexin-V and PE (ElabscienceCat No.: E-CK-A211) and analyzed with flow cytometry.

### Colony formation assay with stable transfected cells

HepG2 cells were transfected with different plasmid constructs (null plasmid, pT7-V5-SBP-C1-HshnRNP E1, and V5-SBP-KH-1/2/3–only versions). Stable clones were generated by G418 selection for 14 days. After one round selection, cells were detached from the plate and 100 cells per well were seeded in a 6-well plate (for each experiment). Cells were grown for 14 days in conditioned media. Then, cells were fixed with 4% paraformaldehyde and stained with crystal violet. The number of colonies was counted using ImageJ software.

### Scratch assay

HepG2 cells were directly transfected with different plasmids (null plasmid, pT7-V5-SBP-C1-HshnRNP E1, and V5-SBP-KH-1/2/3–only variants). Post 24 h of transfection, the near-confluent cell monolayer was scratched with sterile 200 μl pipette tips. Images of the scratches were captured after 0, 6-, 12-, 18-, and 24-h postscratching to record the healing of the scratched areas. ImageJ software was used to evaluate/measure the scratch area and accordingly the percentage of scratch/wound recovery was calculated.

### Prediction of hnRNP E1 and p53/p21 transcript interaction and identification of interacting RNA motifs

For predicting hnRNPE1 and p53/p21 transcript interaction, we used catRAPID (http://service.tartaglialab.com/page/catrapid_omics2_group), an online algorithm for estimating the binding propensity of protein–RNA interaction. We went with the catRAPID graphic option which predicted interactions between protein–RNA and was used to evaluate the interaction between hnRNP E1 and p53 5′ & 3′-UTRs, as well as for hnRNP E1 and p21 5′ & 3′-UTRs based on hydrogen bonding, interatomic forces, *etc*. For hnRNP E1, the amino acid sequence of the plasmid pT7-V5-SBP-C1-HshnRNP E1 was used and for p53 and p21, the sequences used are NM_000546.6 and NM_000389.5 respectively. In this entire study, the nucleotide positions are numbered as per the base position of NM_000546.6 and NM_000389.5.

The actual prediction of results is shown as a heat map. The x- and y-axis represent the indices of the RNA and protein sequences, respectively. The interaction scores of the heat map ranged from −3 (‘blue’ color) to +3 (red color) represented as a bar scale at the right of each plot. The total sum represents the overall interaction scores of individual amino acid and nucleotide pairs. For predicted interacting RNA motifs, catRAPID omics v2 was used.

### Statistical analysis

Statistical analyses were performed using GraphPad Prism software version 9. Data are represented as mean ± SD. For the numerical datasets, a 2-tailed unpaired Student *t* test or multiple *t* test were used to compare the two groups. A *p*-value for the statistical significance as indicated in the writings and figures are set as ∗*p* < 0.05, ∗∗*p* < 0.01, ∗∗∗*p* < 0.001, ∗∗∗∗*p* < 0.0001.

## Data availability

All data generated during this study are available within the article and the supplementary information files. Further any extra information regarding the experimental details and data will be available upon reasonable request.

## Supporting information

This article contains supporting information.

## Conflict of interests

The authors declare that they have no conflicts of interest with the contents of this article.

## References

[1] Moore M.J. (2005). From birth to death: the complex lives of eukaryotic mRNAs. Science.

[2] Keene J.D. (2010). Minireview: global regulation and dynamics of ribonucleic acid. Endocrinology.

[3] Ji X., Kong J., Liebhaber S.A. (2011). An RNA-protein complex links enhanced nuclear 3' processing with cytoplasmic mRNA stabilization. EMBO J..

[4] Krecic A.M., Swanson M.S. (1999). hnRNP complexes: composition, structure, and function. Curr. Opin. Cell Biol..

[5] Han S.P., Tang Y.H., Smith R. (2010). Functional diversity of the hnRNPs: past, present and perspectives. Biochem. J..

[6] Han N., Li W., Zhang M. (2013). The function of the RNA-binding protein hnRNP in cancer metastasis. J. Cancer Res. Ther..

[7] Dreyfuss G., Matunis M.J., Piñol-Roma S., Burd C.G. (1993). hnRNP proteins and the biogenesis of mRNA. Annu. Rev. Biochem..

[8] Patel S.J., Protchenko O., Shakoury-Elizeh M., Baratz E., Jadhav S., Philpott C.C. (2021). The iron chaperone and nucleic acid-binding activities of poly(rC)-binding protein 1 are separable and independently essential. Proc. Natl. Acad. Sci. U. S. A..

[9] Zhang X., Di C., Chen Y., Wang J., Su R., Huang G. (2020). Multilevel regulation and molecular mechanism of poly (rC)-binding protein 1 in cancer. FASEB J..

[10] Guo J., Jia R. (2018). Splicing factor poly(rC)-binding protein 1 is a novel and distinctive tumor suppressor. J. Cell. Physiol..

[11] Pillai M.R., Chacko P., Kesari L.A., Jayaprakash P.G., Jayaram H.N., Antony A.C. (2003). Expression of folate receptors and heterogeneous nuclear ribonucleoprotein E1 in women with human papillomavirus mediated transformation of cervical tissue to cancer. J. Clin. Pathol..

[12] Zhang Z.Z., Shen Z.Y., Shen Y.Y., Zhao E.H., Wang M., Wang C.J. (2015). HOTAIR long noncoding RNA promotes gastric cancer metastasis through suppression of poly r(C)-Binding protein (PCBP) 1. Mol. Cancer Ther..

[13] Zhang Y., Meng L., Xiao L., Liu R., Li Z., Wang Y.L. (2018). The RNA-binding protein PCBP1 functions as a tumor suppressor in prostate cancer by inhibiting mitogen activated protein kinase 1. Cell Physiol. Biochem..

[14] Liu Y., Gai L., Liu J., Cui Y., Zhang Y., Feng J. (2015). Expression of poly(C)-binding protein 1 (PCBP1) in NSCLC as a negative regulator of EMT and its clinical value. Int. J. Clin. Exp. Pathol..

[15] Chkheidze A.N., Liebhaber S.A. (2003). A novel set of nuclear localization signals determine distributions of the alphaCP RNA-binding proteins. Mol. Cell. Biol..

[16] Gamarnik A.V., Andino R. (1997). Two functional complexes formed by KH domain containing proteins with the 5' noncoding region of poliovirus RNA. RNA.

[17] Valverde R., Edwards L., Regan L. (2008). Structure and function of KH domains. FEBS J..

[18] Sidiqi M., Wilce J.A., Vivian J.P., Porter C.J., Barker A., Leedman P.J. (2005). Structure and RNA binding of the third KH domain of poly(C)-binding protein 1. Nucleic Acids Res..

[19] Musco G., Stier G., Joseph C., Castiglione Morelli M.A., Nilges M., Gibson T.J. (1996). Three-dimensional structure and stability of the KH domain: molecular insights into the fragile X syndrome. Cell.

[20] Dejgaard K., Leffers H. (1996). Characterisation of the nucleic-acid-binding activity of KH domains. Different properties of different domains. Eur. J. Biochem..

[21] Grelet S., Howe P.H. (2019). hnRNP E1 at the crossroads of translational regulation of epithelial-mesenchymal transition. J. Cancer Metastasis Treat..

[22] Zhang W., Zhang S., Guan W., Huang Z., Kong J., Huang C. (2020). Poly C binding protein 1 regulates p62/SQSTM1 mRNA stability and autophagic degradation to repress tumor progression. Front. Genet..

[23] Zheng Y., Zhou Z., Wei R., Xiao C., Zhang H., Fan T. (2022). The RNA-binding protein PCBP1 represses lung adenocarcinoma progression by stabilizing DKK1 mRNA and subsequently downregulating β-catenin. J. Transl. Med..

[24] Lin L., Li H., Shi D., Liu Z., Wei Y., Wang W. (2022). Depletion of C12orf48 inhibits gastric cancer growth and metastasis via up-regulating Poly r(C)-Binding protein (PCBP) 1. BMC Cancer.

[25] Brown A.S., Mohanty B.K., Howe P.H. (2016). Identification and characterization of an hnRNP E1 translational silencing motif. Nucleic Acids Res..

[26] Hussey G.S., Chaudhury A., Dawson A.E., Lindner D.J., Knudsen C.R., Wilce M.C. (2011). Identification of an mRNP complex regulating tumorigenesis at the translational elongation step. Mol. Cell..

[27] Chaudhury A., Hussey G.S., Howe P.H. (2011). 3'-UTR-mediated post-transcriptional regulation of cancer metastasis: beginning at the end. RNA Biol..

[28] Huo L.R., Zhong N. (2008). Identification of transcripts and translatants targeted by overexpressed PCBP1. Biochim. Biophys. Acta.

[29] Huo L.R., Ju W., Yan M., Zou J.H., Yan W., He B. (2010). Identification of differentially expressed transcripts and translatants targeted by knock-down of endogenous PCBP1. Biochim. Biophys. Acta.

[30] Huang S., Luo K., Jiang L., Zhang X.D., Lv Y.H., Li R.F. (2021). PCBP1 regulates the transcription and alternative splicing of metastasis-related genes and pathways in hepatocellular carcinoma. Sci. Rep..

[31] Hanahan D. (2022). Hallmarks of cancer: new dimensions. Cancer Discov..

[32] Agostini F., Zanzoni A., Klus P., Marchese D., Cirillo D., Tartaglia G.G. (2013). catRAPID omics: a web server for large-scale prediction of protein-RNA interactions. Bioinformatics.

[33] Armaos A., Colantoni A., Proietti G., Rupert J., Tartaglia G.G. (2021). catRAPID omics v2.0: going deeper and wider in the prediction of protein-RNA interactions. Nucleic Acids Res..

[34] Gong X., Tian M., Cao N., Yang P., Xu Z., Zheng S. (2021). Circular RNA circEsyt2 regulates vascular smooth muscle cell remodeling via splicing regulation. J. Clin. Invest..

[35] Giles K.M., Daly J.M., Beveridge D.J., Thomson A.M., Voon D.C., Furneaux H.M. (2003). The 3'-untranslated region of p21WAF1 mRNA is a composite cis-acting sequence bound by RNA-binding proteins from breast cancer cells, including HuR and poly(C)-binding protein. J. Biol. Chem..

[36] Yoga Y.M., Traore D.A., Sidiqi M., Szeto C., Pendini N.R., Barker A. (2012). Contribution of the first K-homology domain of poly(C)-binding protein 1 to its affinity and specificity for C-rich oligonucleotides. Nucleic Acids Res..

[37] Gu S., Jin L., Zhang F., Sarnow P., Kay M.A. (2009). Biological basis for restriction of microRNA targets to the 3' untranslated region in mammalian mRNAs. Nat. Struct. Mol. Biol..

[38] Ji X., Kong J., Liebhaber S.,A. (2003). In vivo association of the stability control protein αCP with actively translating mRNAs. Molecular and cellular biology. Mol. Cell. Biol..

[39] Chaudhury A., Chander P., Howe P.H. (2010). Heterogeneous nuclear ribonucleoproteins (hnRNPs) in cellular processes: focus on hnRNP E1's multifunctional regulatory roles. RNA.

[40] Kiliszek A., Rypniewski W., Błaszczyk L. (2023). Exploring structural determinants and the role of nucleolin in formation of the long-range interactions between untranslated regions of p53 mRNA. RNA.

[41] Chen J., Kastan M.B. (2010). 5'-3'-UTR interactions regulate p53 mRNA translation and provide a target for modulating p53 induction after DNA damage. Genes Dev..

[42] Swiatkowska A., Zydowicz P., Sroka J., Ciesiołka J. (2016). The role of the 5' terminal region of p53 mRNA in the p53 gene expression. Acta Biochim. Pol..

[43] Swiatkowska A., Dutkiewicz M., Zydowicz-Machtel P., Szpotkowska J., Janecki D.M., Ciesiołka J. (2019). Translational control in *p53* expression: the role of 5'-Terminal region of p53 mRNA. Int. J. Mol. Sci..

[44] Wang X., Guo J., Che X., Jia R. (2019). PCBP1 inhibits the expression of oncogenic STAT3 isoform by targeting alternative splicing of STAT3 exon 23. Int. J. Biol. Sci..

[45] Ostermeier M., Benkovic S.J. (2000). Evolution of protein function by domain swapping. Adv. Protein Chem..

[46] Messih M.A., Chitale M., Bajic V.B., Kihara D., Gao X. (2012). Protein domain recurrence and order can enhance prediction of protein functions. Bioinformatics.

[47] Lahtvee P.J., Sánchez B.J., Smialowska A., Kasvandik S., Elsemman I.E., Gatto F. (2017). Absolute quantification of protein and mRNA abundances demonstrate variability in gene-specific translation efficiency in yeast. Cell Syst..

[48] Mazor K.M., Dong L., Mao Y., Swanda R.V., Qian S.B., Stipanuk M.H. (2018). Effects of single amino acid deficiency on mRNA translation are markedly different for methionine versus leucine. Sci. Rep..

[49] Krissinel E. (2007). On the relationship between sequence and structure similarities in proteomics. Bioinformatics.

[50] Wright C.M., Wright R.C., Eshleman J.R., Ostermeier M. (2011). A protein therapeutic modality founded on molecular regulation. Proc. Natl. Acad. Sci. U. S. A..

[51] Mukherjee A.G., Wanjari U.R., Gopalakrishnan A.V., Bradu P., Biswas A., Ganesan R. (2023). Evolving strategies and application of proteins and peptide therapeutics in cancer treatment. Biomed. Pharmacother..

[52] Biswas A., Treadaway J., Tellinghuisen T.L. (2016). Interaction between nonstructural proteins NS4B and NS5A is essential for proper NS5A localization and hepatitis C virus RNA replication. J. Virol..

[53] Qi D., Scholthof K.B. (2008). A one-step PCR-based method for rapid and efficient site-directed fragment deletion, insertion, and substitution mutagenesis. J. Virol. Methods.

[54] Shimakami T., Yamane D., Jangra R.K., Kempf B.J., Spaniel C., Barton D.J. (2012). Stabilization of hepatitis C virus RNA by an Ago2-miR-122 complex. Proc. Natl. Acad. Sci. U. S. A..

[55] Somasekharan S.P., Zhang F., Saxena N., Huang J.N., Kuo I.C., Low C. (2020). G3BP1-linked mRNA partitioning supports selective protein synthesis in response to oxidative stress. Nucleic Acids Res..

[56] Itahana Y., Zhang J., Göke J., Vardy L.A., Han R., Iwamoto K. (2016). Histone modifications and p53 binding poise the p21 promoter for activation in human embryonic stem cells. Sci. Rep..

[57] Karamysheva Z.N., Tikhonova E.B., Grozdanov P.N., Huffman J.C., Baca K.R., Karamyshev A. (2018). Polysome profiling in Leishmania, human cells and mouse testis. J. Vis. Exp..

[58] Dixit U., Pandey A.K., Mishra P., Sengupta A., Pandey V.N. (2016). Staufen1 promotes HCV replication by inhibiting protein kinase R and transporting viral RNA to the site of translation and replication in the cells. Nucleic Acids Res..

[59] Okuda K.S., Keyser M.S., Gurevich D.B., Sturtzel C., Mason E.A., Paterson S. (2021). Live-imaging of endothelial Erk activity reveals dynamic and sequential signalling events during regenerative angiogenesis. eLife.

