# Supplementary material for: The RNA-binding protein hnRNP E1 regulates p53 and p21 translation via KH1 and KH2 domain interactions with 3′ UTR C-rich motifs
Source: J Biol Chem. 2025 Dec 12;302(2):111042. doi: 10.1016/j.jbc.2025.111042 (PMC12816904; doi:10.1016/j.jbc.2025.111042)

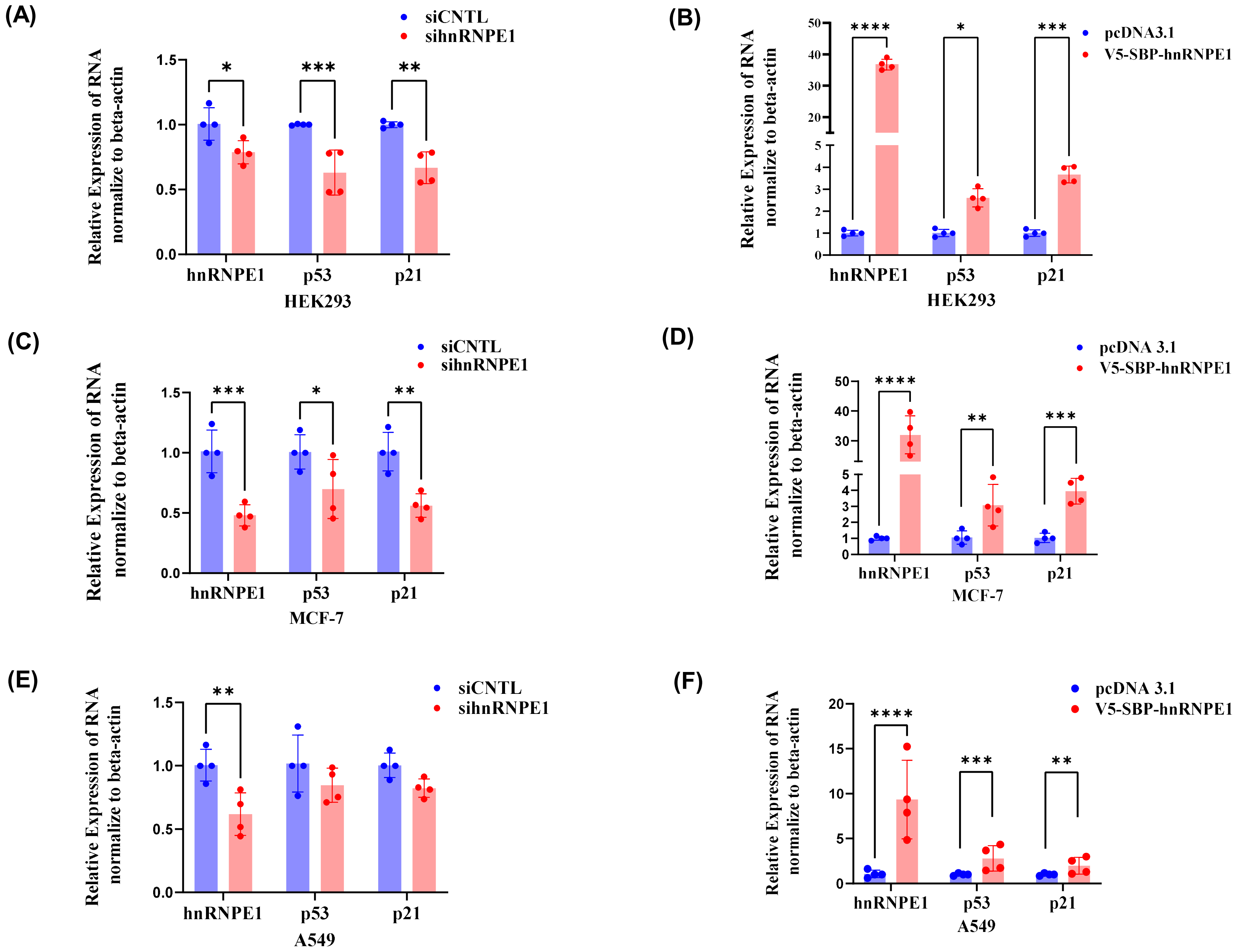

Supplementary figure 1:

hnRNP E1 silencing leads to reduction in p53 and p21 RNA levels (A, C, and E), whereas hnRNP E1 overexpression leads to induction in their RNA levels (B, D, and F) in HEK 293, MCF-7 and A549 cell lines, as quantified by real-time PCR. *p<0.05, **p<0.01, ***p<0.001, ****p<0.0001. All the experiments were done in triplicate.

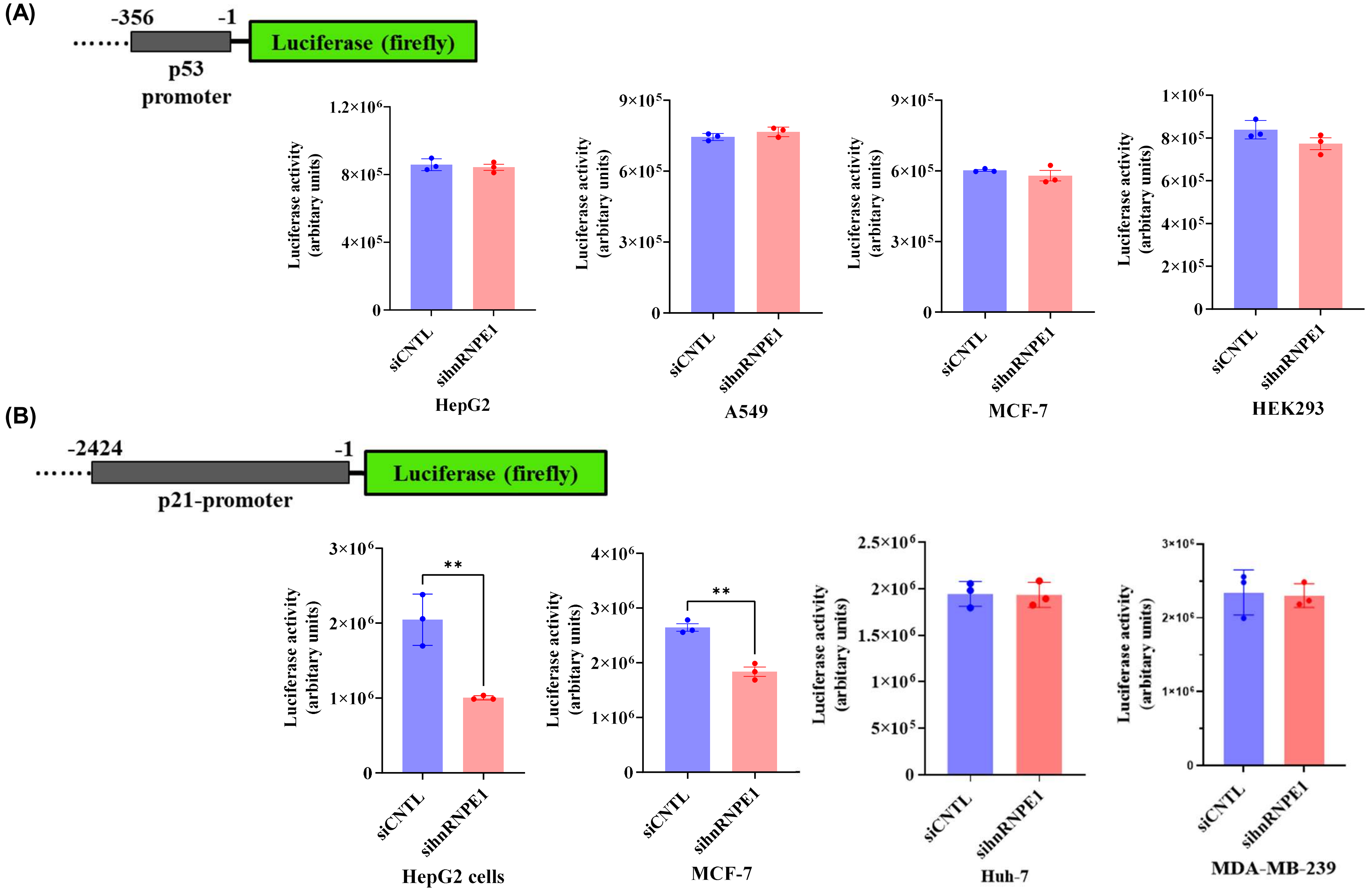

Supplementary figure 2:

hnRNP E1 does not influence p53 and p21 promoter activity. **(A)** The functional region of the plasmid pGL2-356 bp (for p53 promoter activity assay) and the luciferase assay readouts (bar graphs) in hnRNP E1-silenced conditions are shown in the figure and the assays were done in HepG2, A549, MCF-7 and Hek293 cells. **(B)** The functional region of the plasmid pGL2-p21 promoter-Luc (for p21 promoter activity assay) and the luciferase assay readouts (bar graphs) in hnRNP E1-silenced conditions are shown in the figure and the assays were done in HepG2, MCF-7, Huh-7 and MDA-MB-239 cells. *$p<0.05$, **$p<0.01$. The data originated from three independent experiments.

(A)

```
RNA_motifs      position_start  position_end    matched_sequence
Q15365|PCBP1|760|16854432|SpliceAid-F,Q15365|PCBP1|93|11827469|RBPDB,Q15365|PCBP1|774|12011088|SpliceAid-
F,Q15365|PCBP1|763|7607214|SpliceAid-F,Q15365|PCBP1|776|17928403|       1373    1379    CCCTCCC
Q15365|PCBP1|775|12011088|SpliceAid-F   2079    2085    TTTTTTT
Q15365|PCBP1|775|12011088|SpliceAid-F   2080    2086    TTTTTTT
Q15365|PCBP1|775|12011088|SpliceAid-F   2081    2087    TTTTTTT
Q15365|PCBP1|775|12011088|SpliceAid-F   2082    2088    TTTTTTT
Q15365|PCBP1|775|12011088|SpliceAid-F   2083    2089    TTTTTTT
Q15365|PCBP1|775|12011088|SpliceAid-F   2084    2090    TTTTTTT
Q15365|PCBP1|775|12011088|SpliceAid-F   2085    2091    TTTTTTT
Q15365|PCBP1|775|12011088|SpliceAid-F   2086    2092    TTTTTTT
Q15365|PCBP1|775|12011088|SpliceAid-F   2087    2093    TTTTTTT
Q15365|PCBP1|775|12011088|SpliceAid-F   2088    2094    TTTTTTT
Q15365|PCBP1|775|12011088|SpliceAid-F   2089    2095    TTTTTTT
Q15365|PCBP1|775|12011088|SpliceAid-F   2090    2096    TTTTTTT
Q15365|PCBP1|760|16854432|SpliceAid-F,Q15365|PCBP1|93|11827469|RBPDB,Q15365|PCBP1|774|12011088|SpliceAid-
F,Q15365|PCBP1|763|7607214|SpliceAid-F,Q15365|PCBP1|776|17928403|       2433    2439    CCCCACC
Q15365|PCBP1|M177_0.6|23846655|cisBP-RNA,Q15365|PCBP1|ccwwhcc|10075886|RBPMap,Q15365|PCBP1|749|17609276|SpliceAid-
F,Q15365|PCBP1|233|31724725|RBNS_ENCODE,Q15365|PCBP1|754|NA|NA  2438    2445    CCCTTCCC
Q15365|PCBP1|M177_0.6|23846655|cisBP-RNA,Q15365|PCBP1|ccwwhcc|10075886|RBPMap,Q15365|PCBP1|749|17609276|SpliceAid-
F,Q15365|PCBP1|233|31724725|RBNS_ENCODE,Q15365|PCBP1|754|NA|NA  433     440     CCCTTCCC
Q15365|PCBP1|760|16854432|SpliceAid-F,Q15365|PCBP1|93|11827469|RBPDB,Q15365|PCBP1|774|12011088|SpliceAid-
F,Q15365|PCBP1|763|7607214|SpliceAid-F,Q15365|PCBP1|776|17928403|       671     677     CCCCACC
```

(B)

```
                                        10
p53 UTR (motif-1; 1373-1379) CCCUCCC
p53 UTR (motif-3; 2433-2439) ...CA..
p53 UTR (motif-4; 2438-2445) ....U..C
p53 UTR (motif-2; 2079-2096) UUU.UUUUUUUUUUUUU
```

(C)

```
RNA_motifs      position_start  position_end    matched_sequence
Q15365|PCBP1|760|16854432|SpliceAid-F,Q15365|PCBP1|93|11827469|RBPDB,Q15365|PCBP1|774|12011088|SpliceAid-
F,Q15365|PCBP1|763|7607214|SpliceAid-F,Q15365|PCBP1|776|17928403|       1398    1404    CCCTCCC
Q15365|PCBP1|760|16854432|SpliceAid-F,Q15365|PCBP1|93|11827469|RBPDB,Q15365|PCBP1|774|12011088|SpliceAid-
F,Q15365|PCBP1|763|7607214|SpliceAid-F,Q15365|PCBP1|776|17928403|       1465    1471    CCCCACC
Q15365|PCBP1|760|16854432|SpliceAid-F,Q15365|PCBP1|93|11827469|RBPDB,Q15365|PCBP1|774|12011088|SpliceAid-
F,Q15365|PCBP1|763|7607214|SpliceAid-F,Q15365|PCBP1|776|17928403|       1717    1723    CCCTCCC
```

(D)

```
                                 10
p21 UTR (motif-1; 1398-1404) CCCUCCC
p21 UTR (motif-2; 1465-1471) ...CA..
p21 UTR (motif-3; 1717-1723) .......
```

Supplementary figure 3:

catRAPID analysis predicts C-rich motifs on 3'-UTRs of p53 and p21 as hnRNP E1 binding sites. **(A)** The analytical output of the catRAPID algorithm of hnRNP E1 binding sites on p53 is shown (http://crg-webservice.s3.amazonaws.com/submissions/2023-02/529510/output/index.html?unlock=12a5cd8f25). **(B)** The alignment of the identified/predicted RNA motif sequences situated on p53 3'-UTR are shown. **(C)** The analytical output of the catRAPID algorithm of hnRNP E1 binding sites on p21 is shown (http://crg-webservice.s3.amazonaws.com/submissions/2024-03/810578/output/index.html?unlock=7c668f69b5). **(D)** The alignment of the identified/predicted RNA motif sequences situated on p21 3'-UTR are shown.

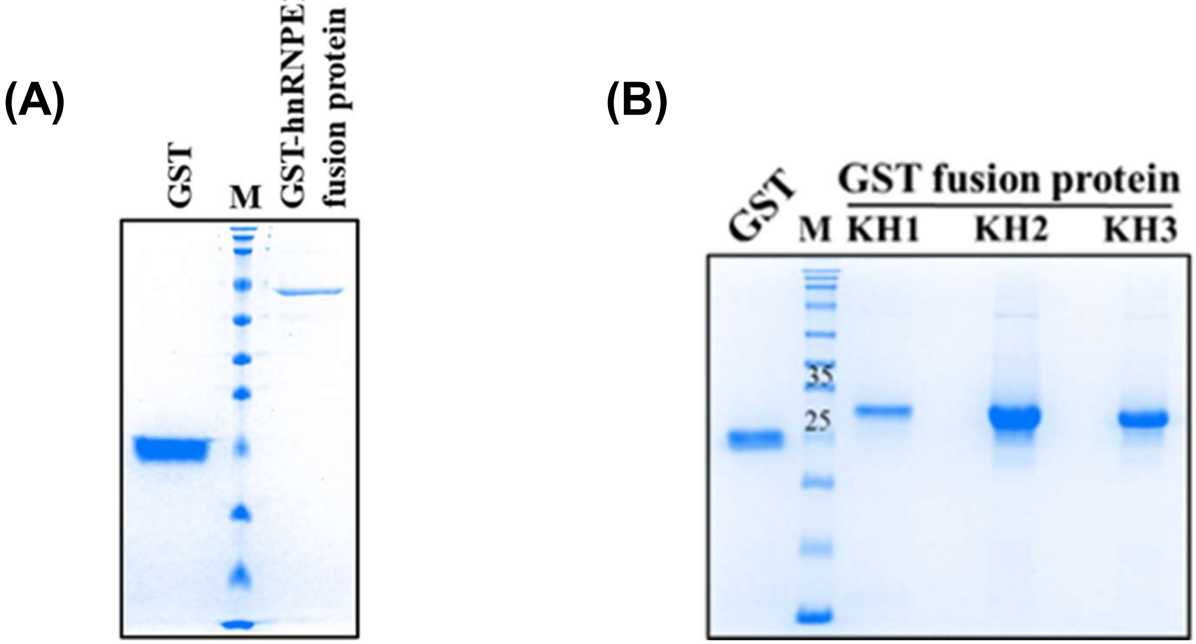

Supplementary figure 4:
(A) Coomassie stained polyacrylamide gel showing the protein bands of purified GST only and GST-hnRNP E1 fusion protein. (**B**) Coomassie stained polyacrylamide gel showing the purified protein bands of GST only and GST-KH domains (1/2/3) of hnRNP E1 proteins.

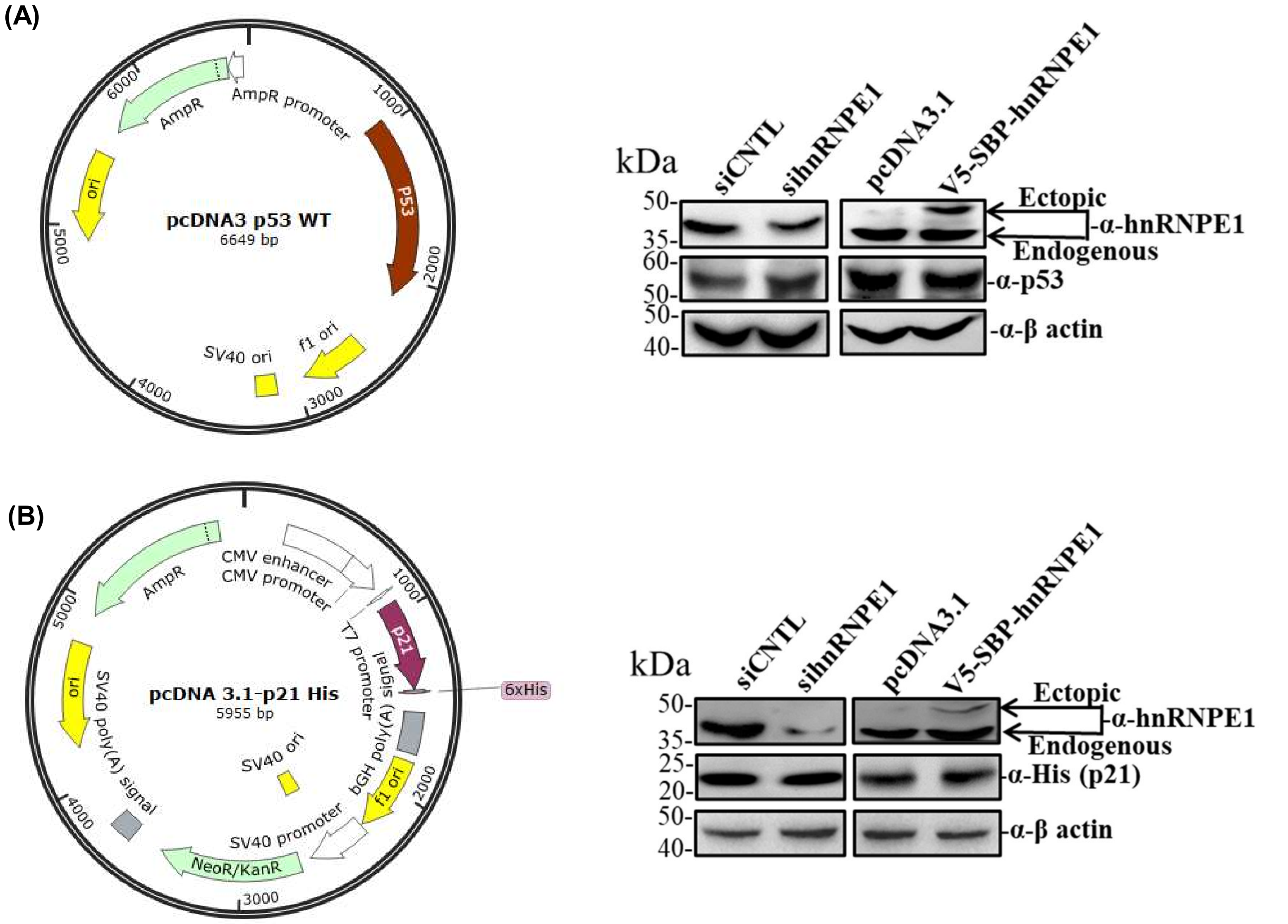

Supplementary figure 5:

hnRNP E1 silencing and overexpression does not affect p53 and p21 cds. **(A)** The plasmid map showing the details of the p53 overexpression system. The western blot panels on the right showing the effect of hnRNP E1 silencing and overexpression on the p53 expression through p53 coding RNA that lacks native UTRs. **(B)** The plasmid map showing the details of the p21 over expression system. The western blot panels on the right showing the effect of hnRNP E1 silencing and over-expression on the p21 expression through p21 coding RNA that lacks native UTRs. The data originated from two independent experiments.

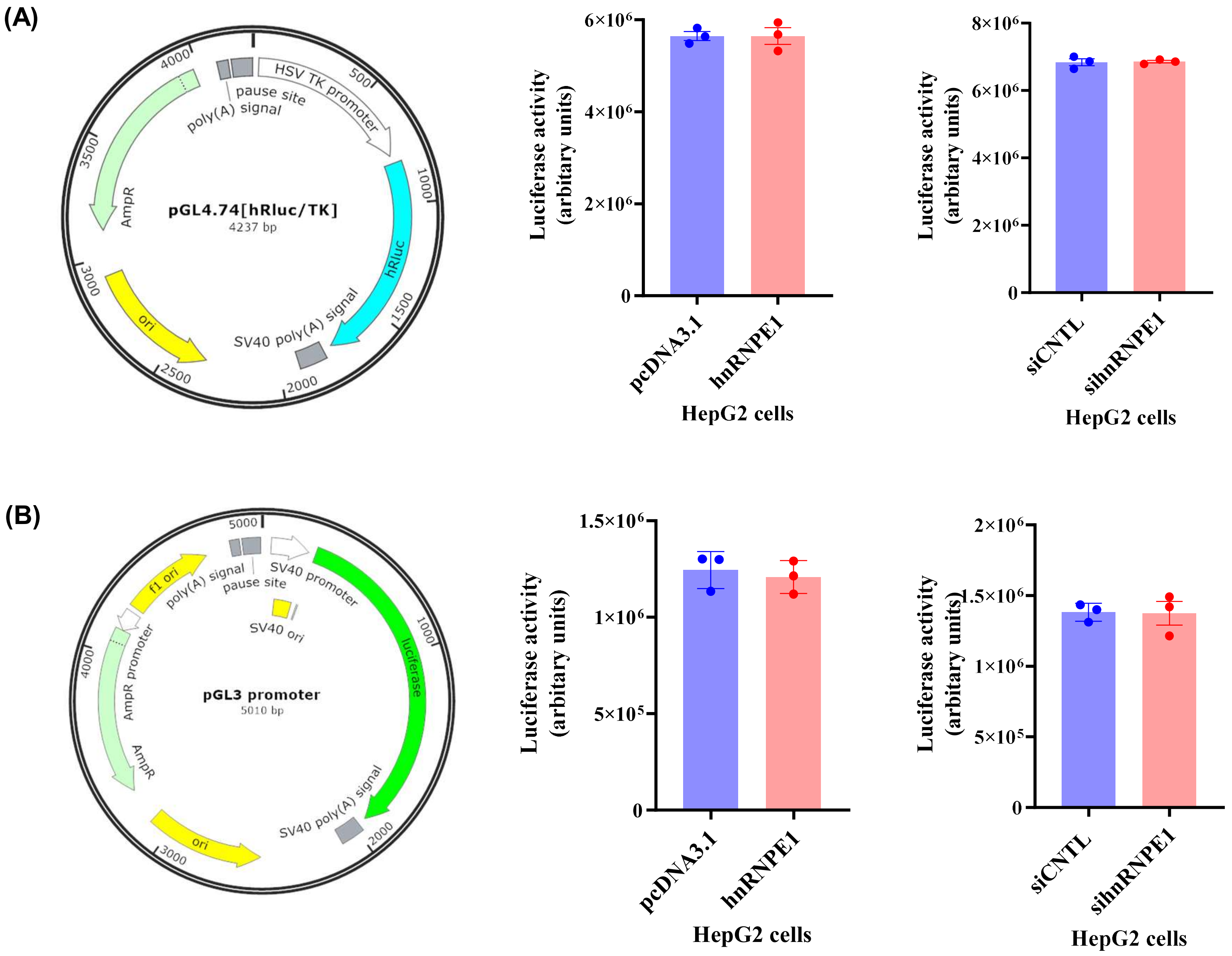

Supplementary figure 6: (A) The plasmid maps of the pGL4.74 [hRluc/TK] is shown on the left and the effect of renilla luciferase expression is represented with bar graphs in both hnRNPE1 overexpressed or silenced cellular conditions with co-transfection based experimental approach. **(B)** The plasmid maps of the pGL3 promoter plasmid map is shown on the left and the effect of firefly luciferase expression is represented with bar graphs in both hnRNPE1 overexpressed or silenced cellular conditions with co-transfection based experimental approach. The data originated from three independent experiments.

(A)

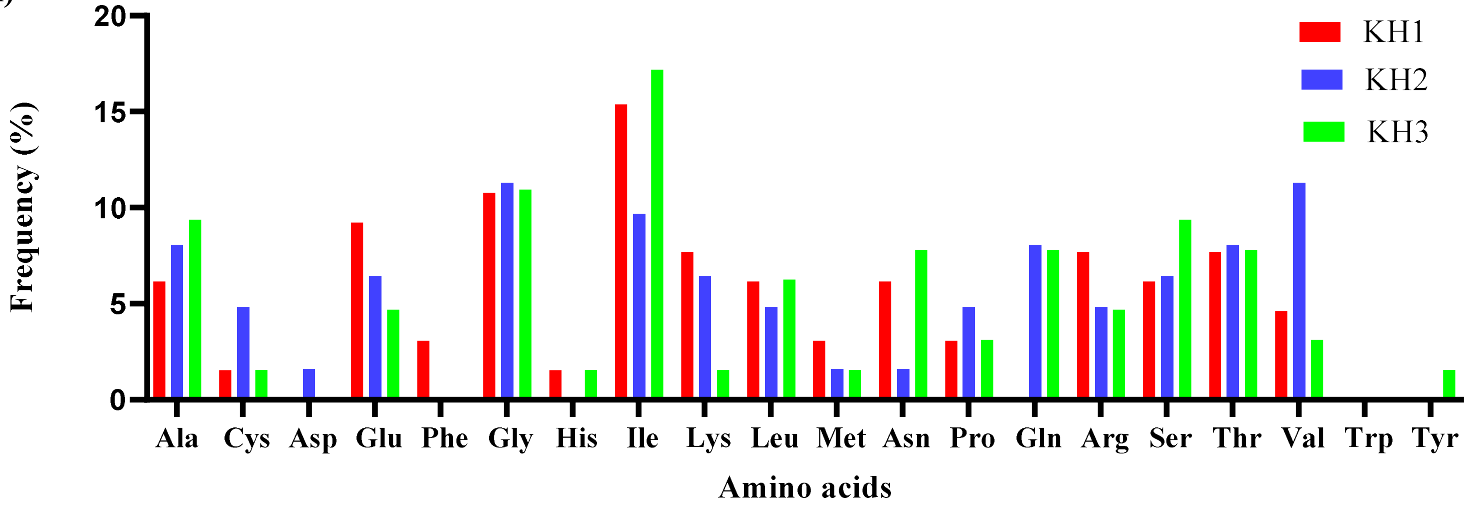

(B)

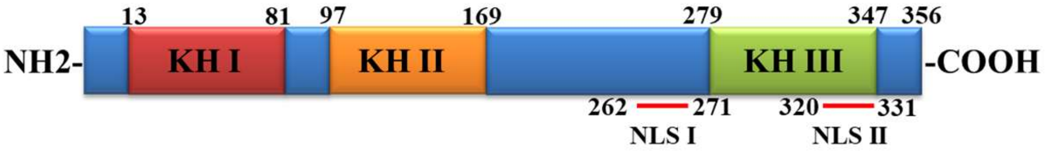

Supplementary figure 7:

(A) The frequency percentages of different amino acids in the three KH domains of hnRNP E1 is shown with the bar diagram. **(B)** Cartoon representing the different structural and functional domains of hnRNP E1 protein.

(A)

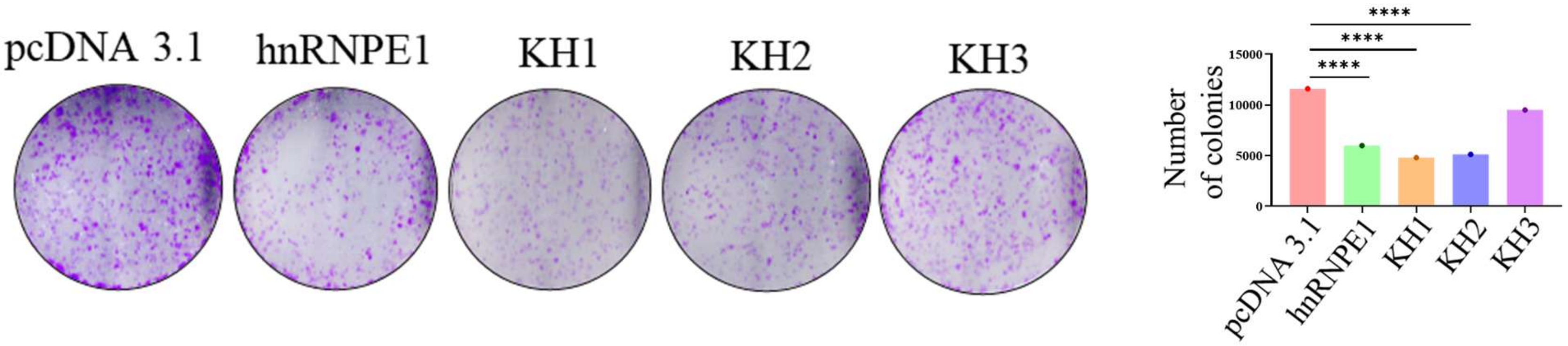

(B)

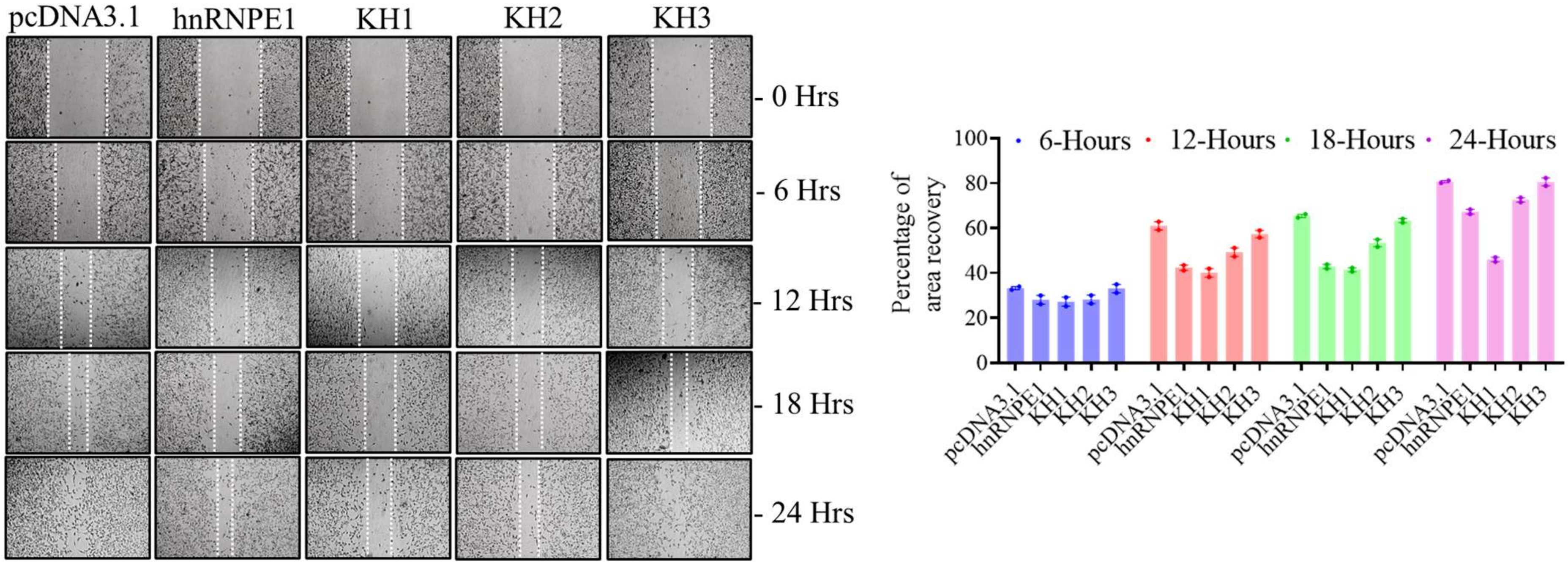

Supplementary figure 8:

KH1 and KH2 domains of hnRNP E1 effectively restricts colony formation and wound healing. **(A)** Images of the wells with crystal violet-stained colonies (HepG2) stably expressing full length hnRNP E1 or individual KH (1/2/3) domains. Bar graph (right) showing the number of colonies as determined by using ImageJ software. **(B)** Scratch assay experiment to assess the wound healing capacity for cells stably expressing full length hnRNP E1 or individual KH (1/2/3) domains. The scratch areas for different experiments were measured using ImageJ software from the images captured at different time points (viz. 0, 6, 12, 18 and 24 hours). The cell free areas were depicted with white dashed lines. Bar graph (right), showing the average percentages of area recovery from two experimental replicates. The percentages of area recovery were calculated by comparing the scratched area with the initial time point i.e. ‘0’ hour.

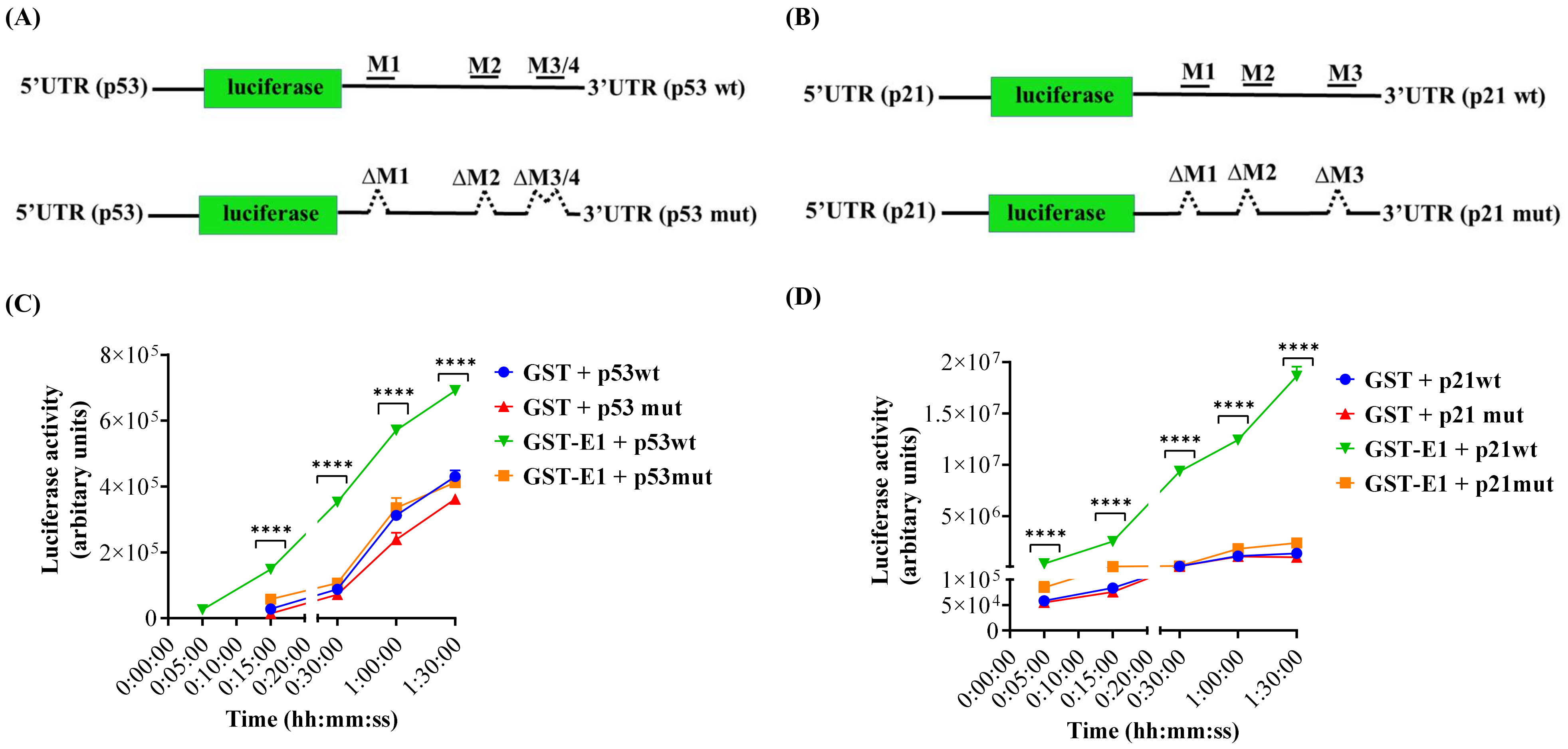

Supplementary figure 9:

RNA motifs (Fig. 3) within 3'-UTRs of p53 and p21 modulates their translation. **(A and B)** Cartoons depicting the wild type (wt) as well as RNA motif deleted (mut) 3'-UTRs of p53 and p21 bearing *in-vitro* transcribed luciferase transcripts respectively. **(C and D)** Line graph showing the *in-vitro* translation efficiency in the form of luciferase signal of *in-vitro* runoff transcripts in presence of both the 5'- and 3'-UTRs of p53 and p21 mRNAs (for both 3'-UTR wildtype as well as motif deletion mutants) in a time dependent manner with purified GST-hnRNP E1 (GST-E1 in the figures) as well as with GST-only (to serve as control) at a concentration of 100 mM. For all the experimental time points (5. 15, 30, 60 and 90 mins) are presented in a linear scale, luciferase activity was measured thrice for all the experimental reactions/groups and the raw values were directly plotted in the graph (*Y*-axis). *p<0.05, **p<0.01, ***p<0.001, ****p<0.0001. The experiments (C and D) are repeated thrice.

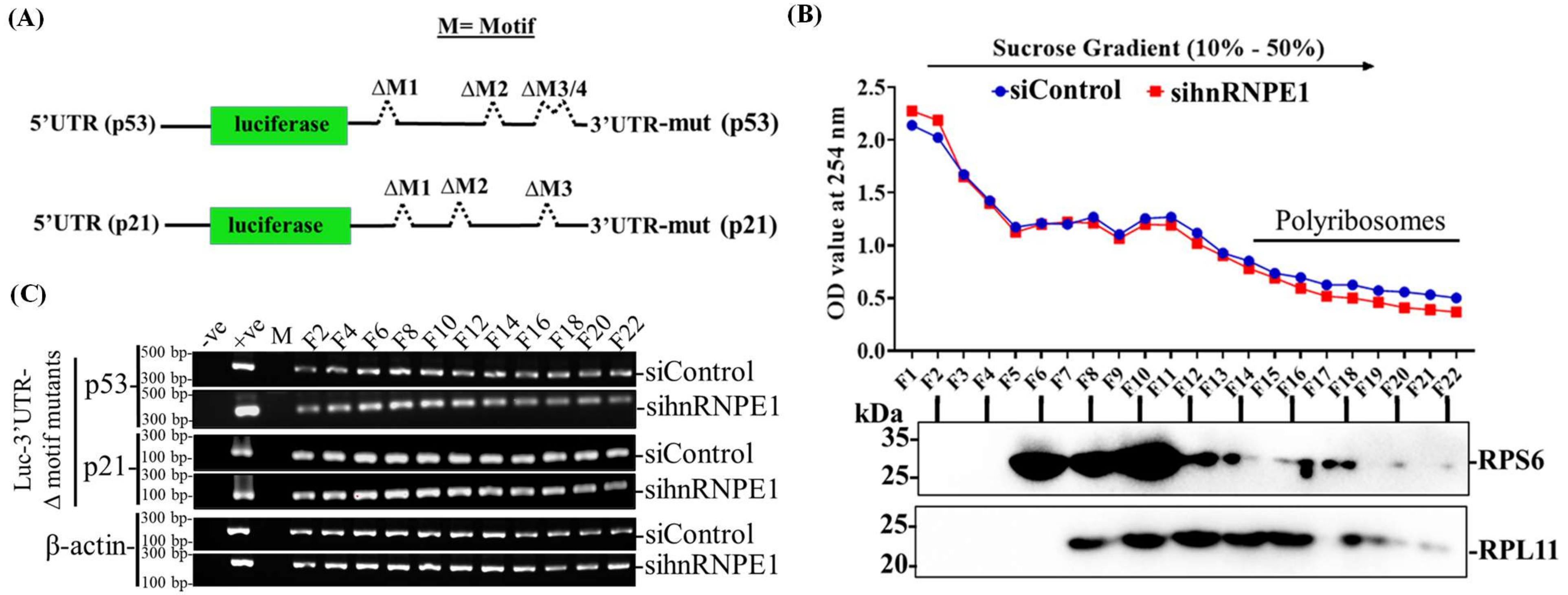

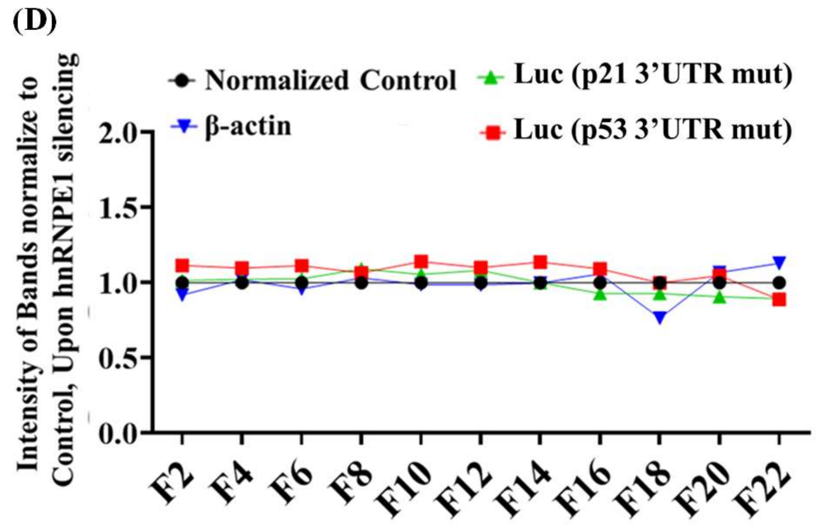

Supplementary figure 10:

RNA motifs (Fig. 3) within 3'-UTRs of p53 and p21 modulates polyribosome formation. **(A)** Cartoon depicting the RNA motif deleted 3'-UTRs (mut) of p53 and p21 generated on p53 5'-UTR-luciferase-3'-UTR expression cassette (145-pGL3ctrl-3' plasmid) and p21 5'-UTR-luciferase-3'-UTR expression cassette (pGL4.74 plasmid). **(B)** The ribosomal profiles of the sucrose density gradient fractions for both p53 and p21 5'-UTR-luciferase-3'-UTR(mut) expressing plasmids co-transfected in hnRNP E1 silenced HepG2 cells (siRNA mediated knockdown) are shown with the fractionation efficiency demonstrated by probing for RPS6 and RPL11 distributions across alternative fractions with western blots. **(C)** Semi-quantitative mid-phase PCR showing the relative abundance of luciferase mRNA (with RNA motif deleted 3'-UTRs of both p53 and p21), as well as β-actin RNA as control in the collected alternative fractions from polyribosome fractionation assay following RNA extraction and cDNA synthesis. **(D)** The PCR amplified band intensities of the amplicons were measured using imageJ software (https://imagej.net/ij/index.html) and the normalized band intensity relative to control (set to 1) for each fractions are plotted in a line graph. The data originated from three independent experiments.

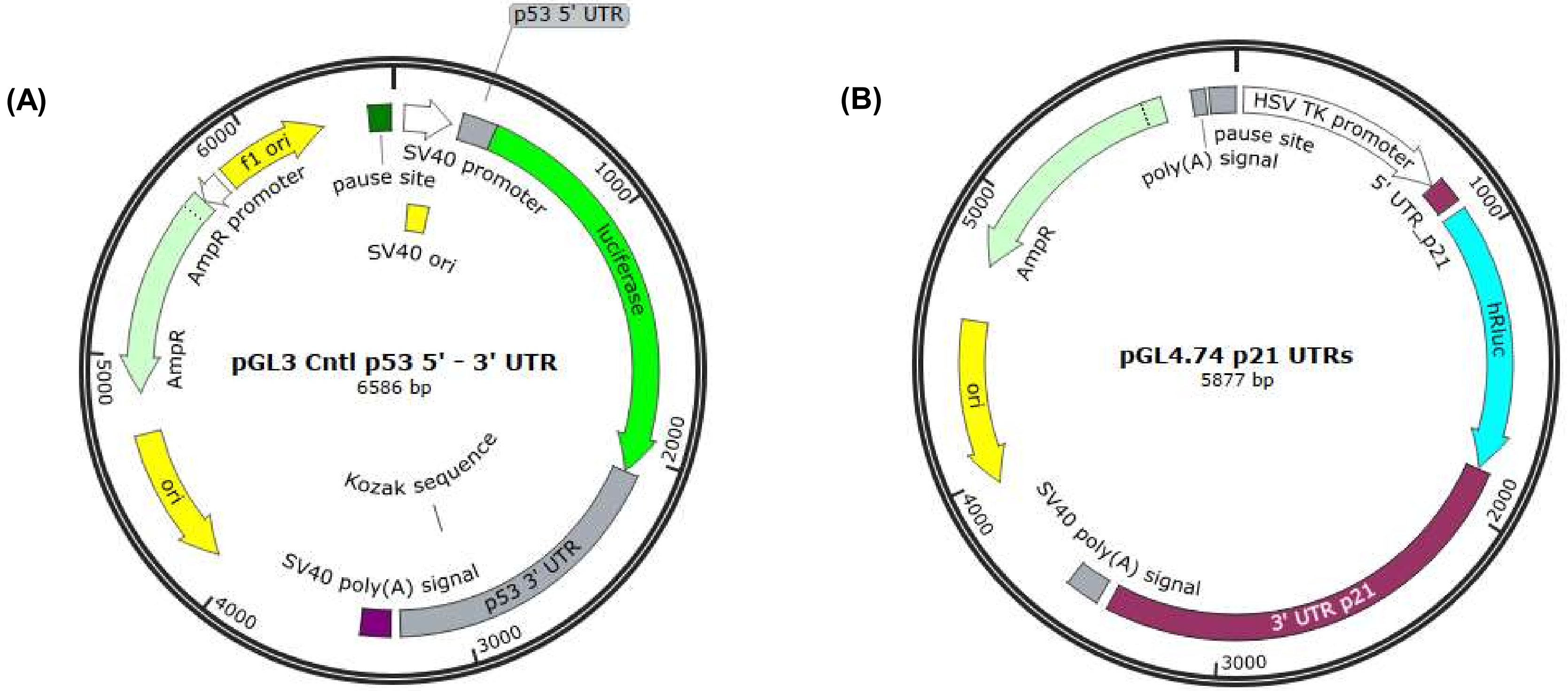

Supplementary figure 11:

The plasmid maps for p53 5’UTR-luciferase-3’UTR expression plasmid i.e. 145-pGL3ctrl-3’ and p21 5’UTR-luciferase-3’UTR expression plasmid are shown in the figures.

Figure 1B **Uncropped western blots**

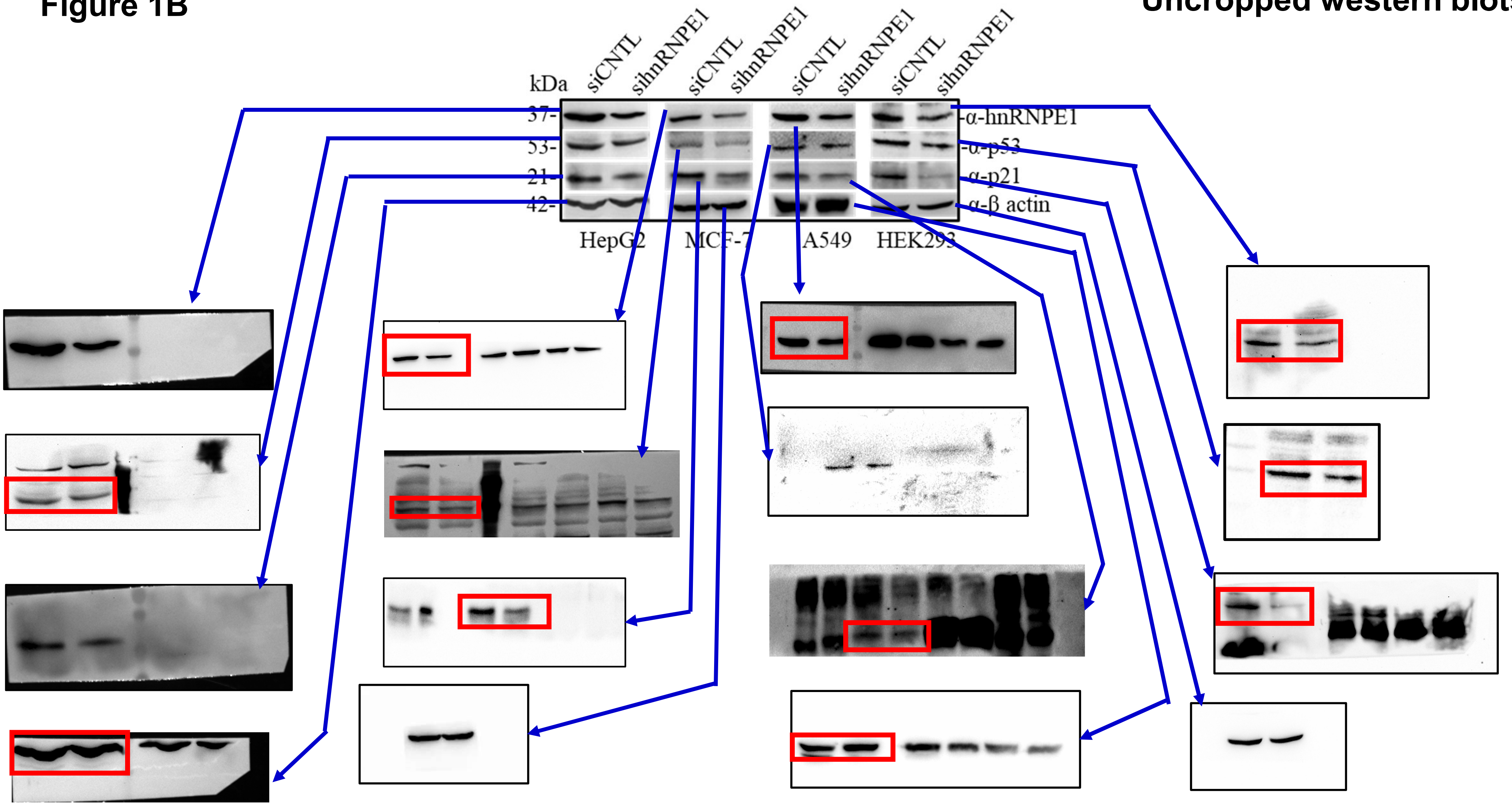

Figure 1D

Uncropped western blots

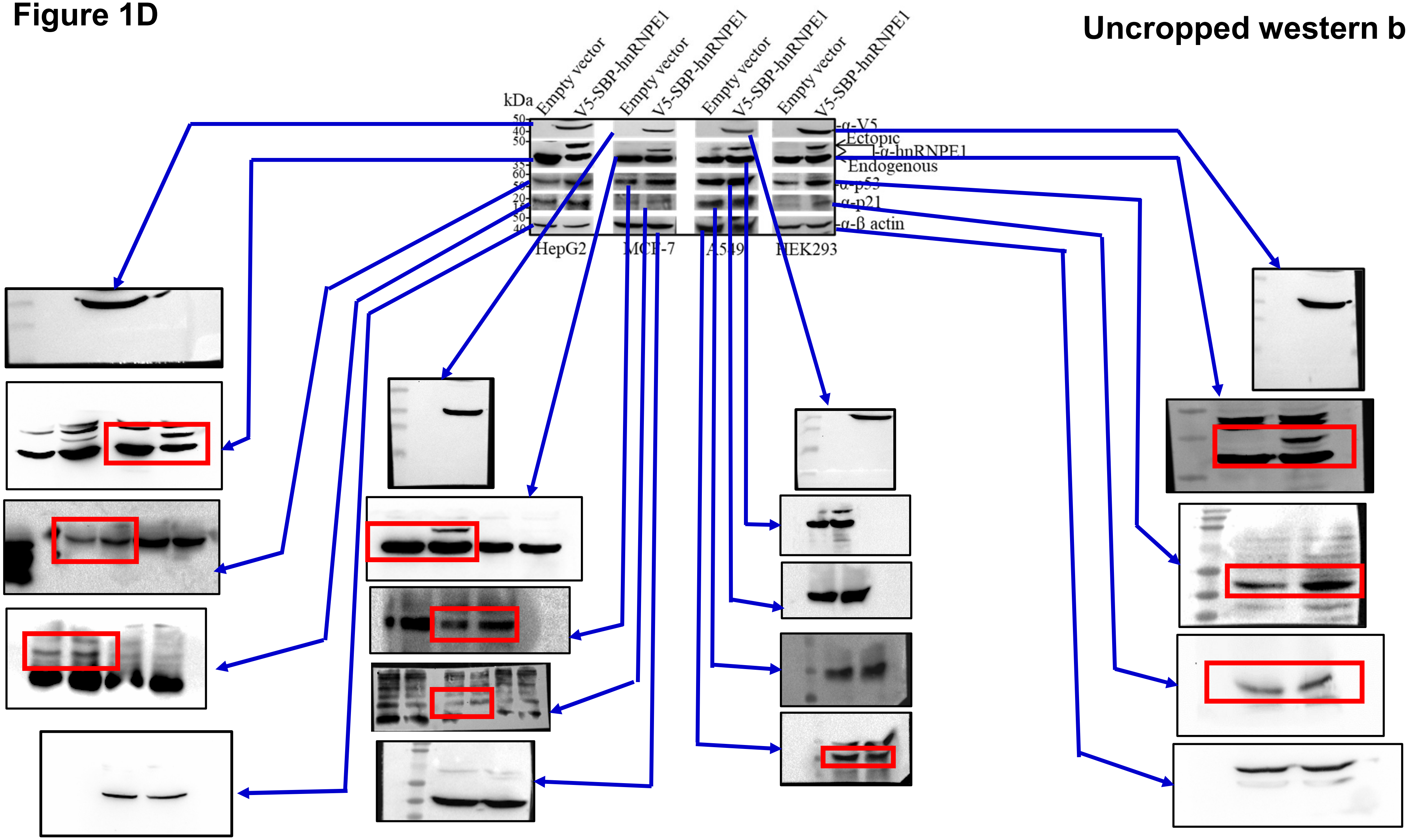

Figure 1F

Uncropped western blots

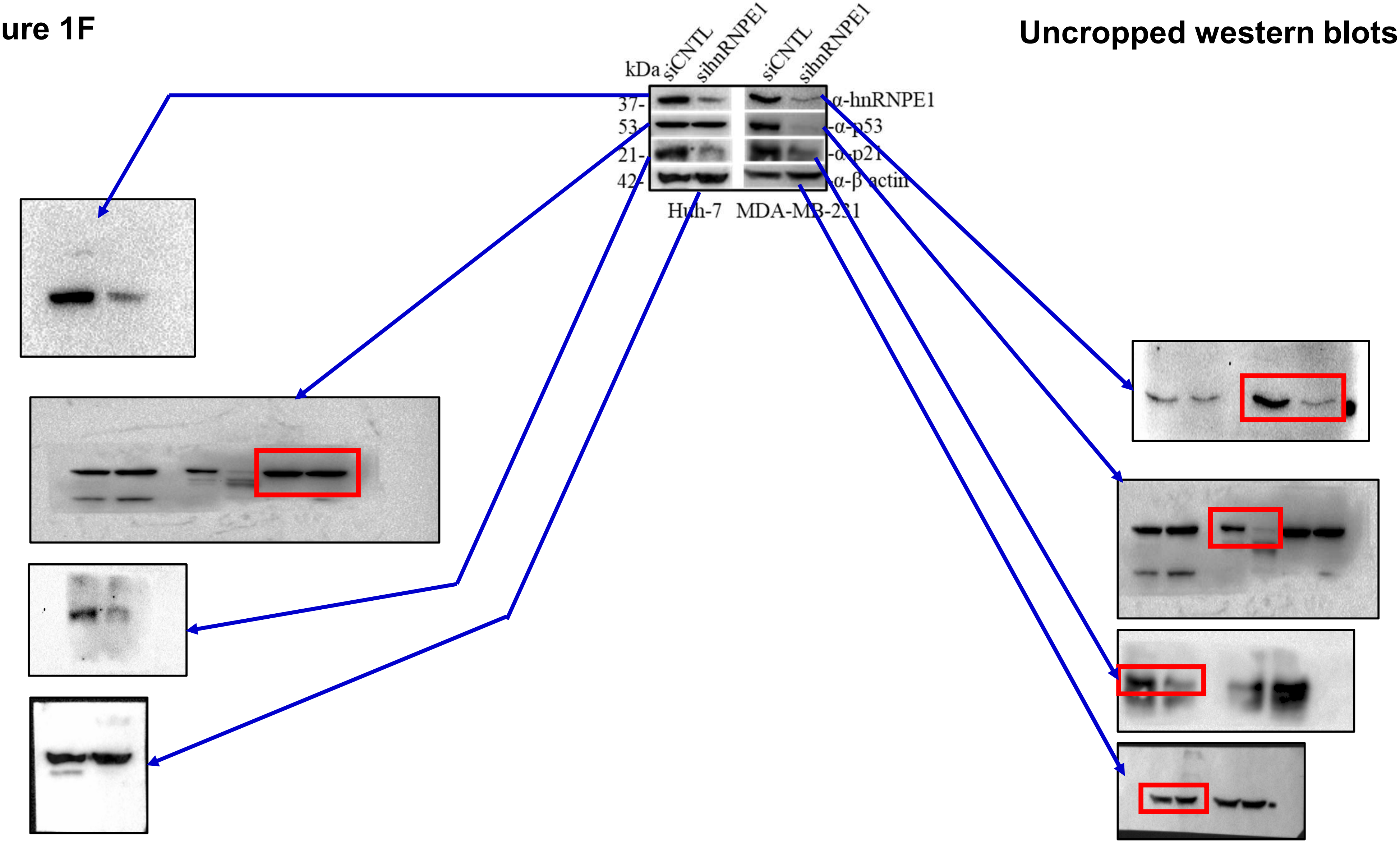

Figure 1H

Uncropped western blots

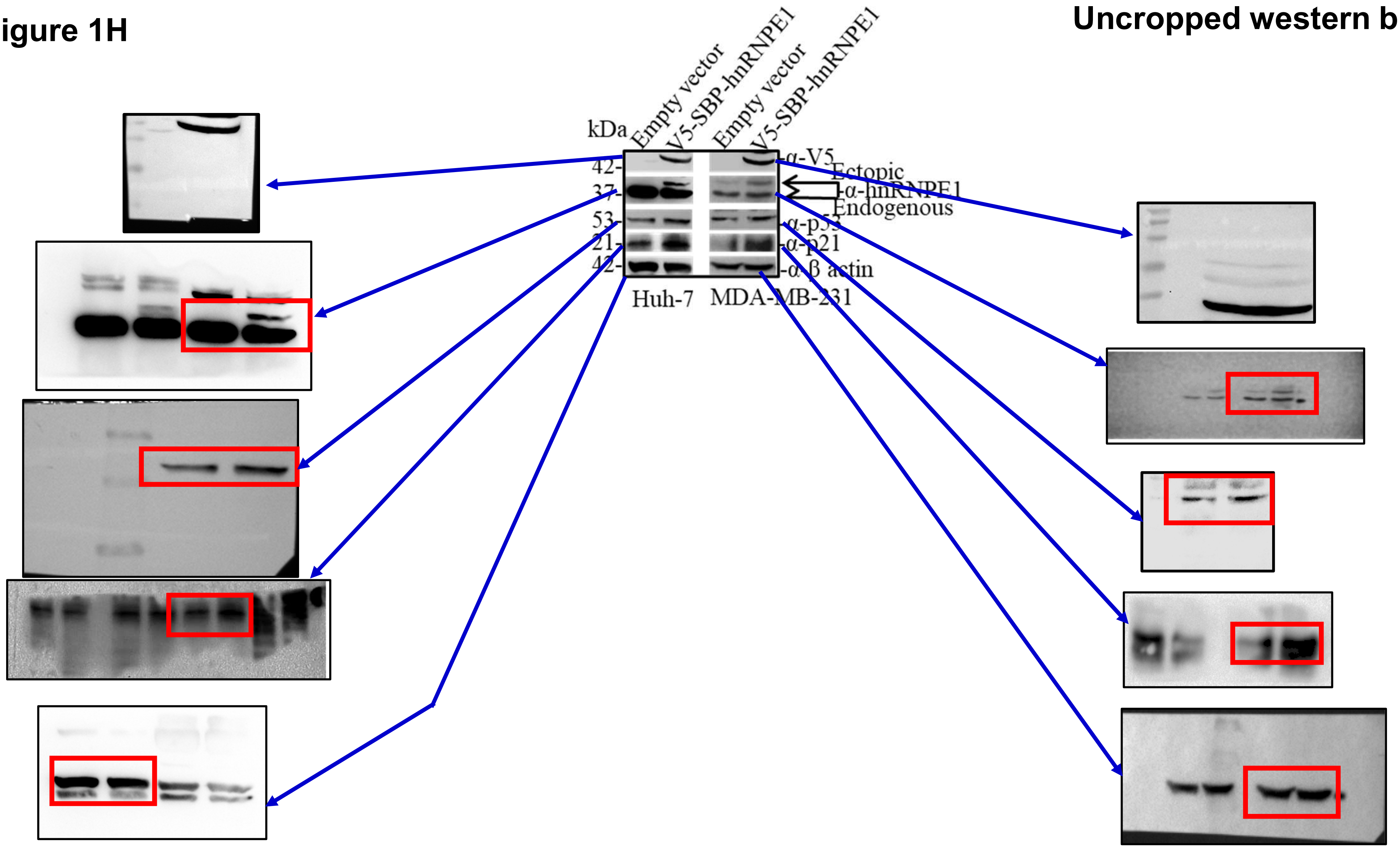

Figure 5A

Uncropped western blots

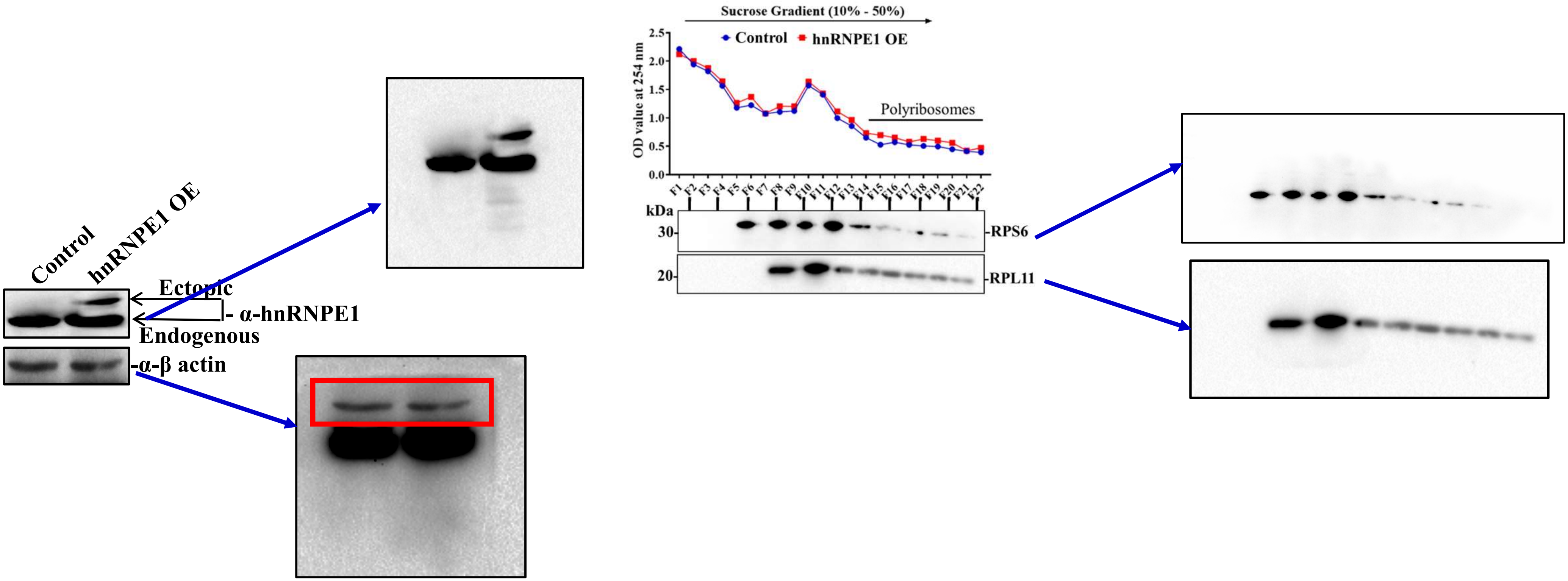

Figure 5B

Uncropped western blots

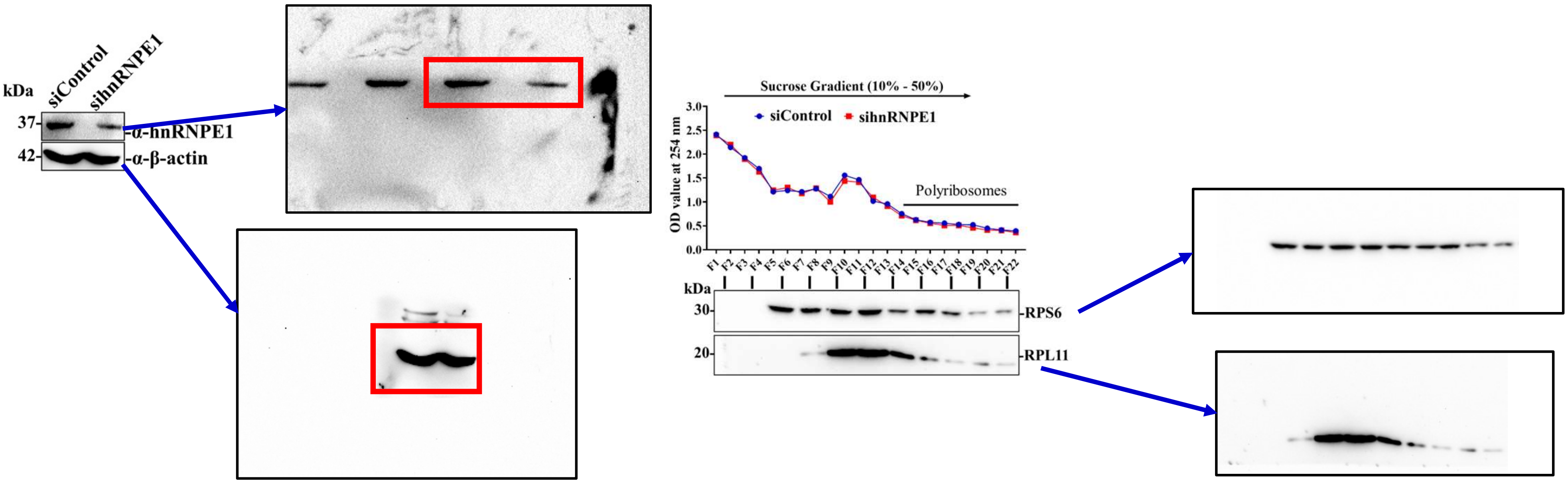

Figure 6F **Uncropped western blots**

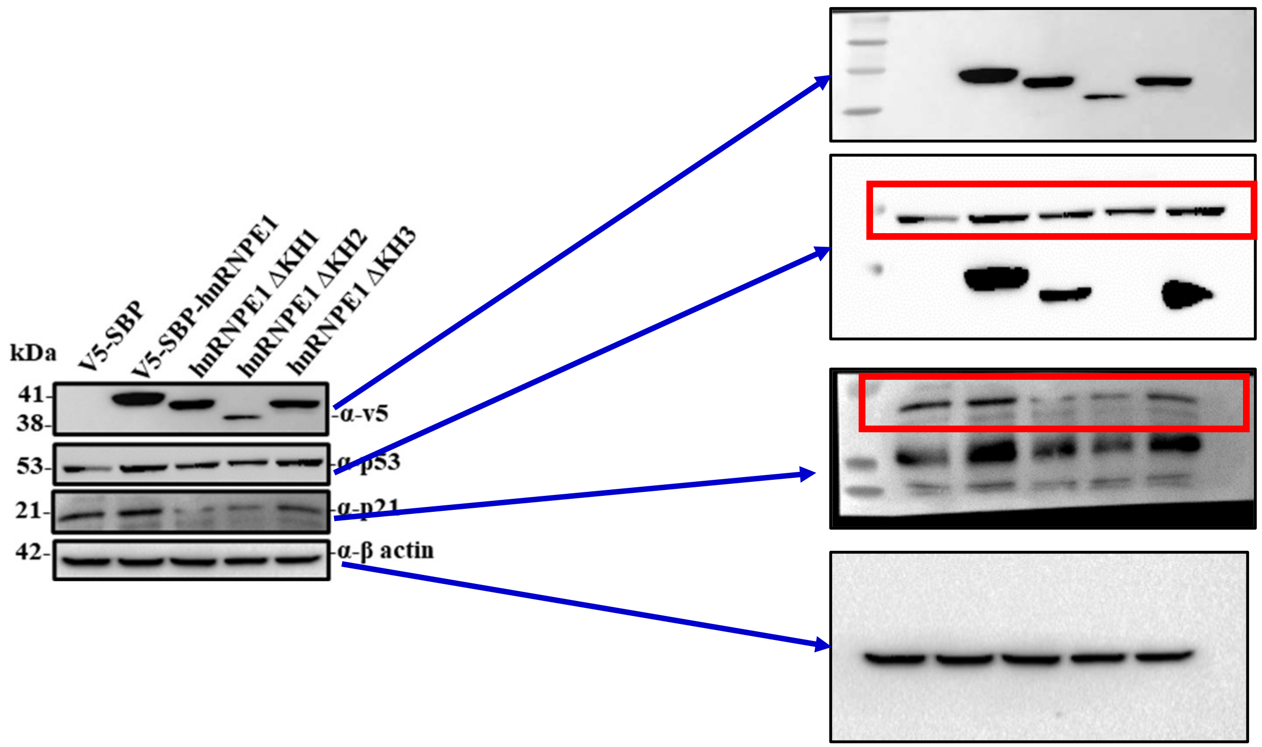

Figure 7B

Uncropped western blots

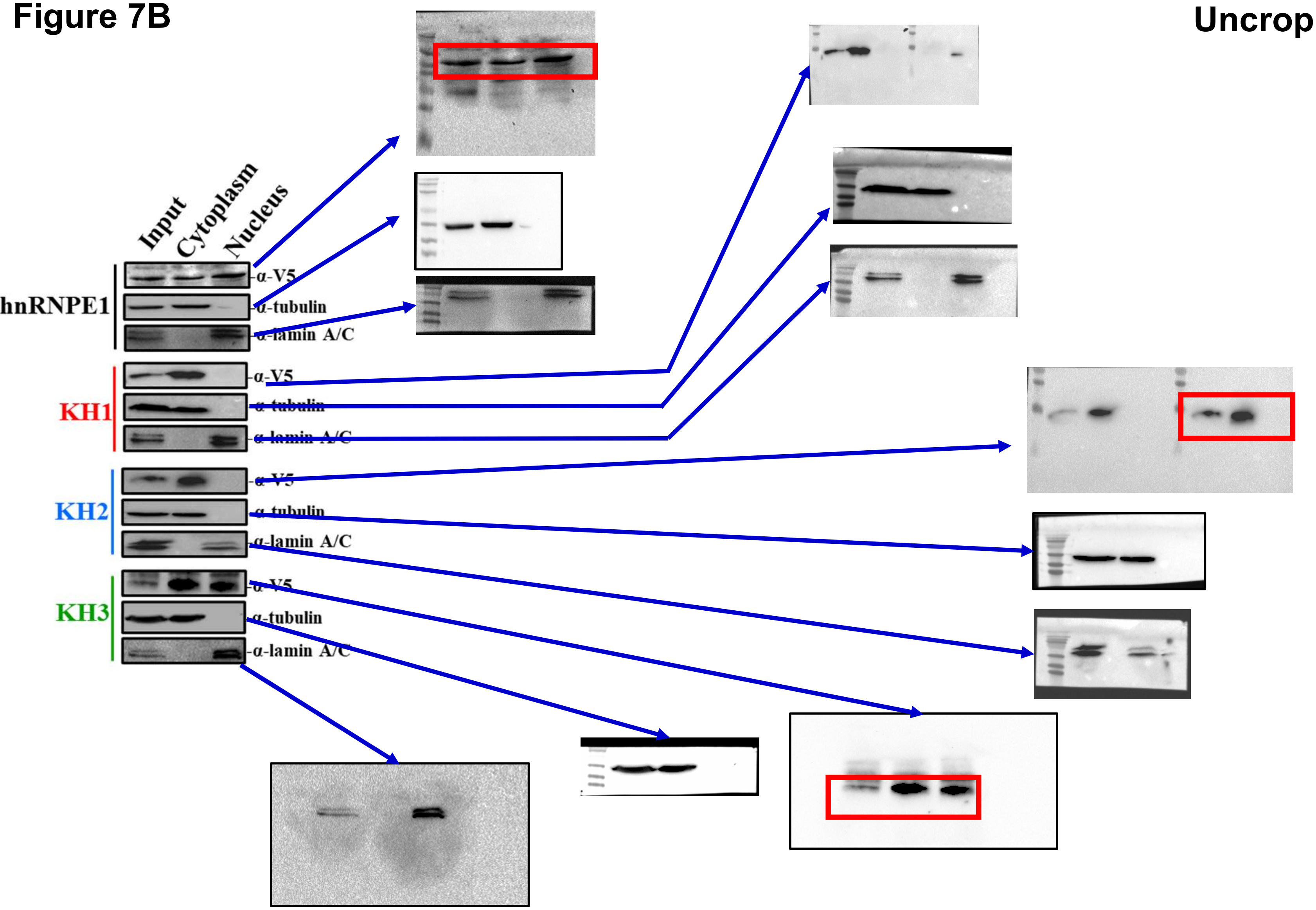

Figure 7E

Uncropped western blots

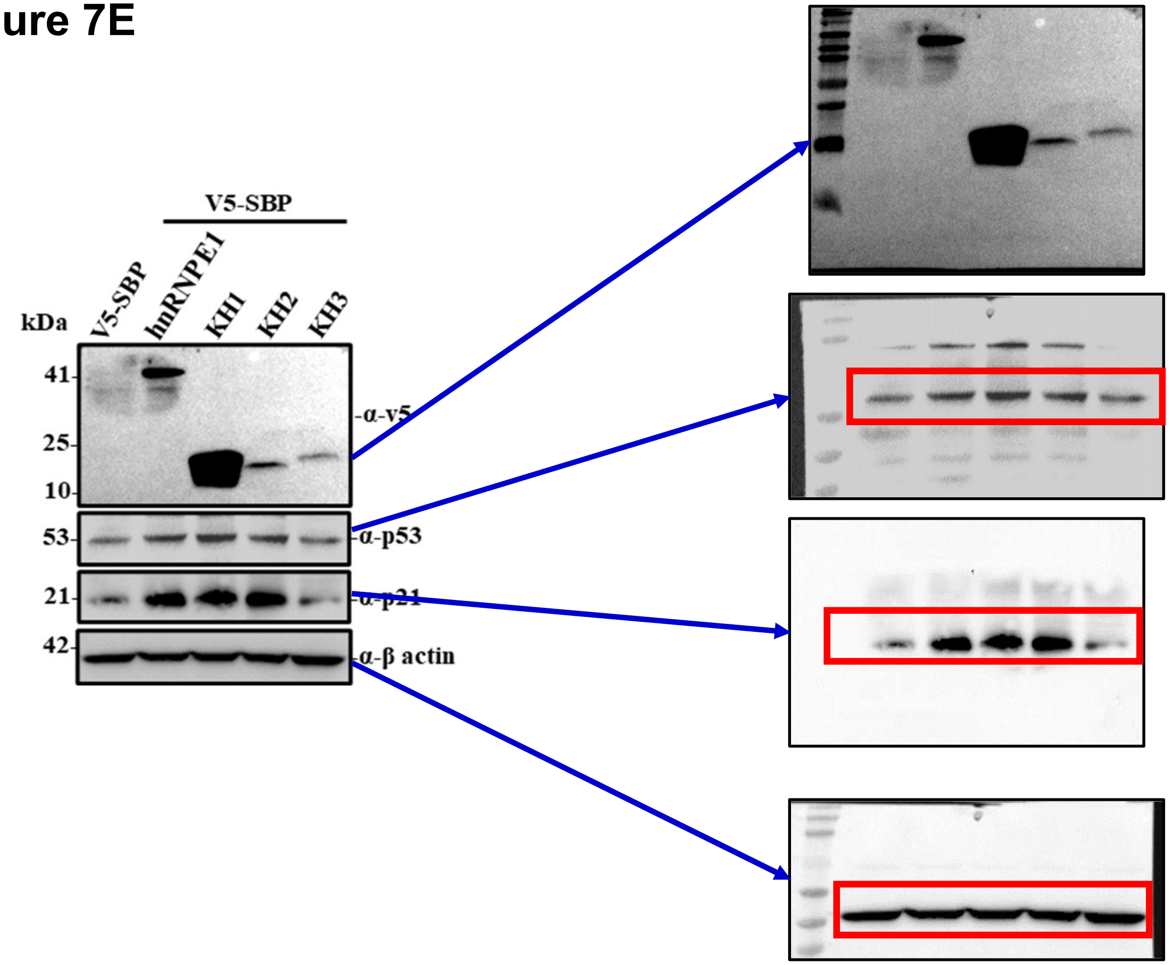

Figure 8D

Uncropped western blots

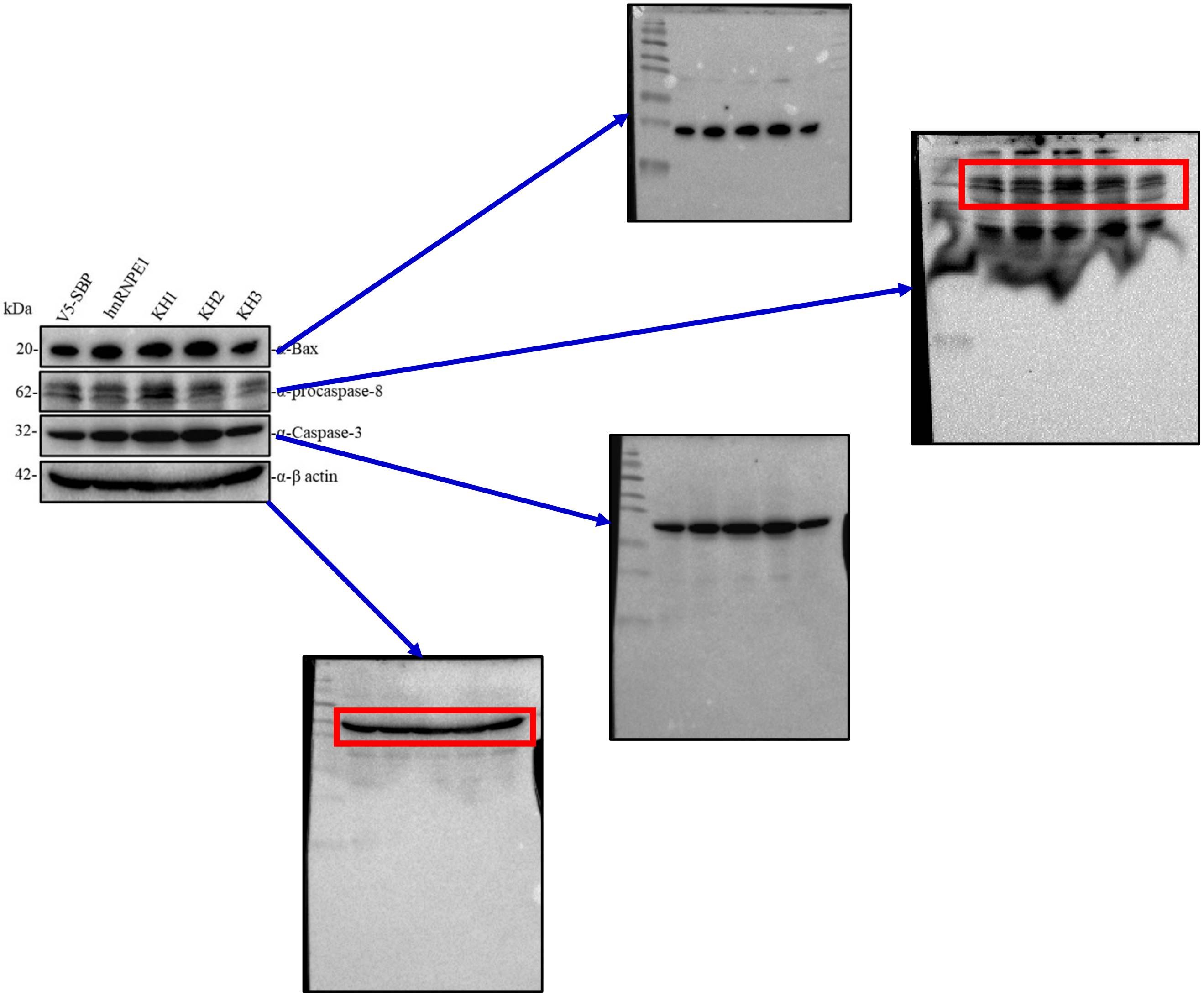

Figure Supplementary 5A

Uncropped western blots

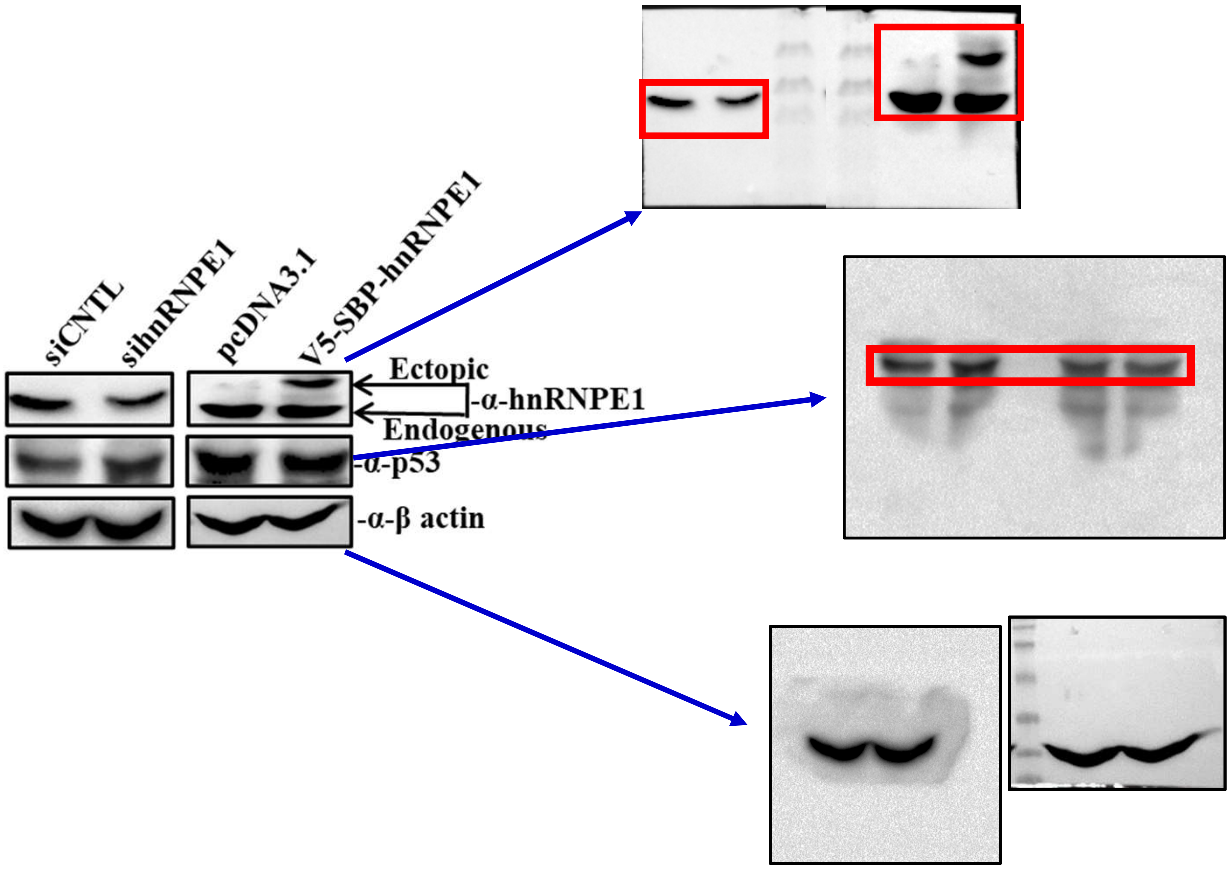

Figure Supplementary 5B **Uncropped western blots**

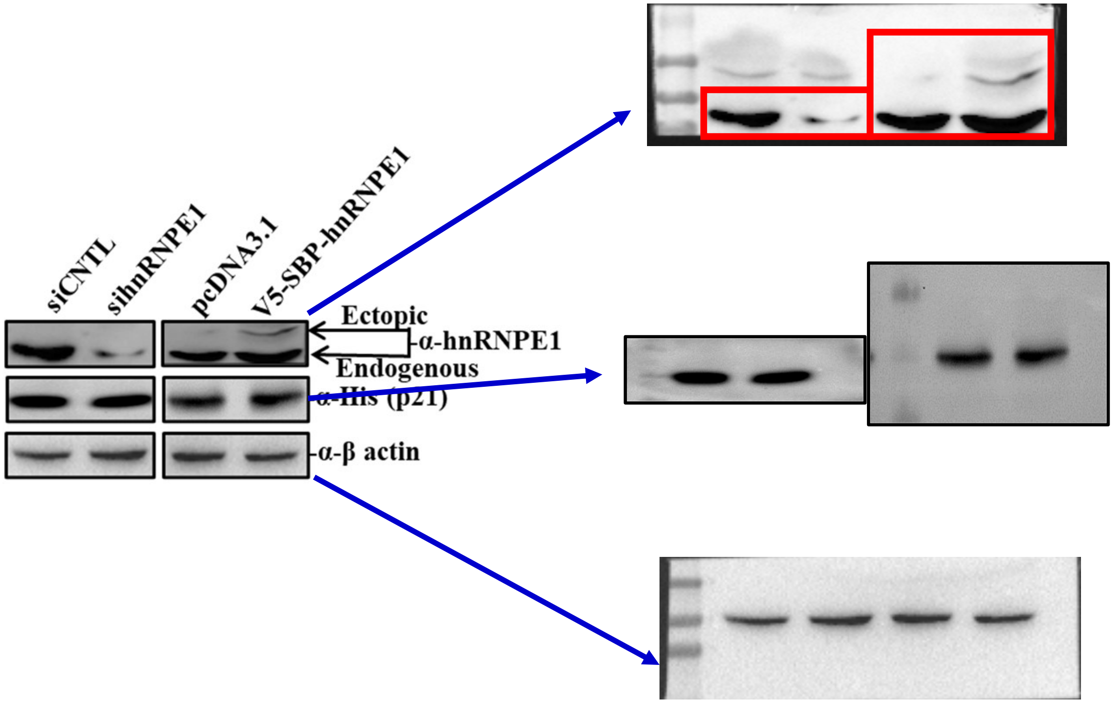

Figure Supplementary 9B

Uncropped western blots

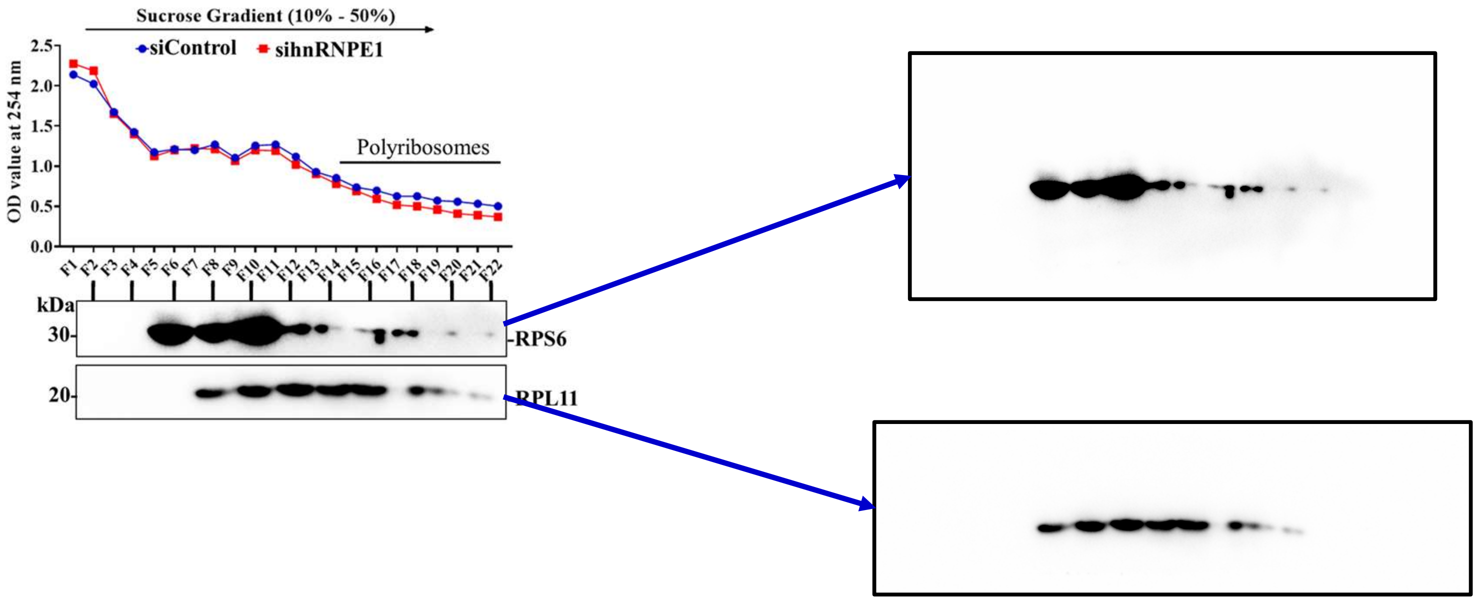

Figure 5C

Uncropped DNA gels

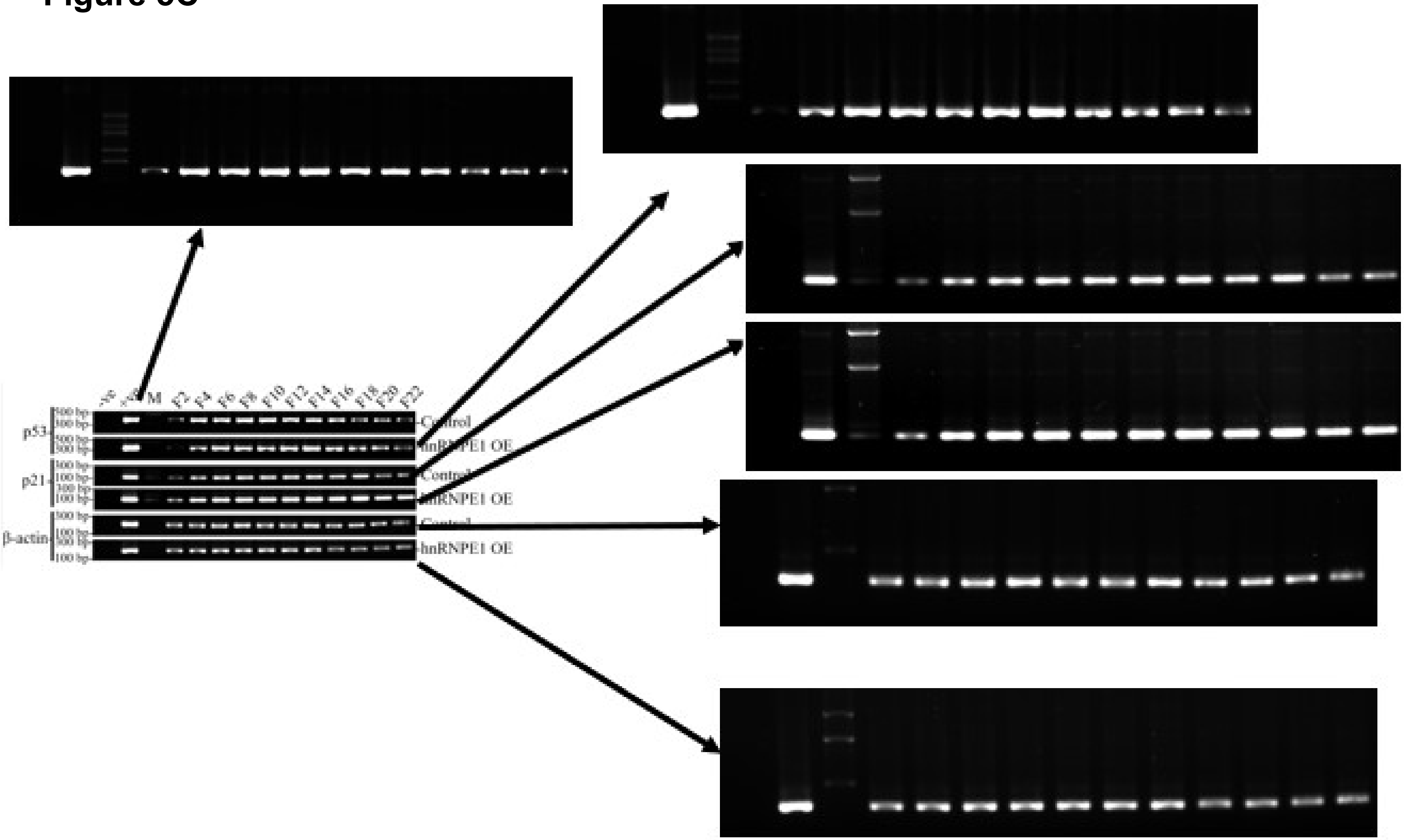

Figure 5D

Uncropped DNA gels

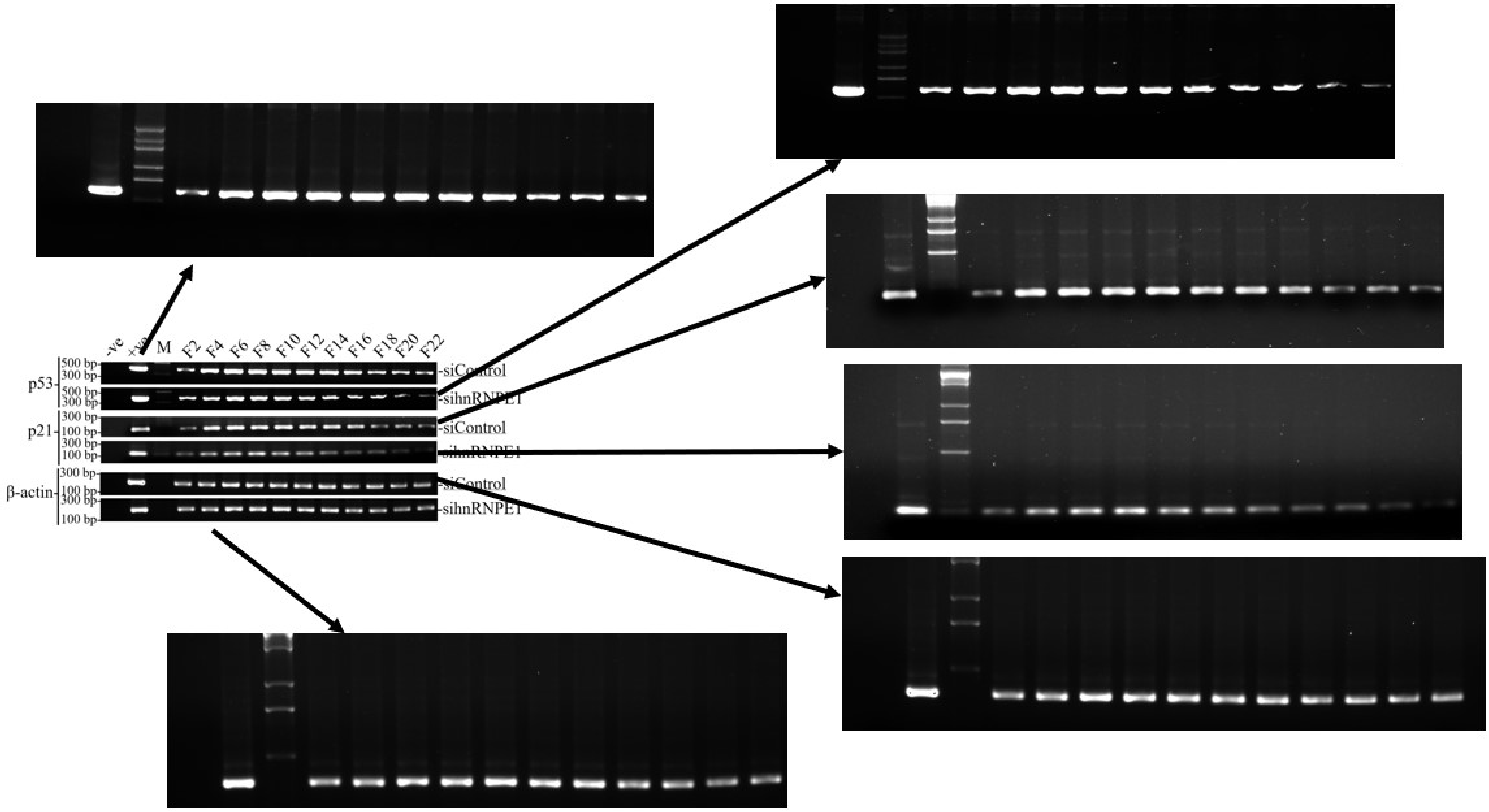

Uncropped DNA gels

Supplementary figure 9C

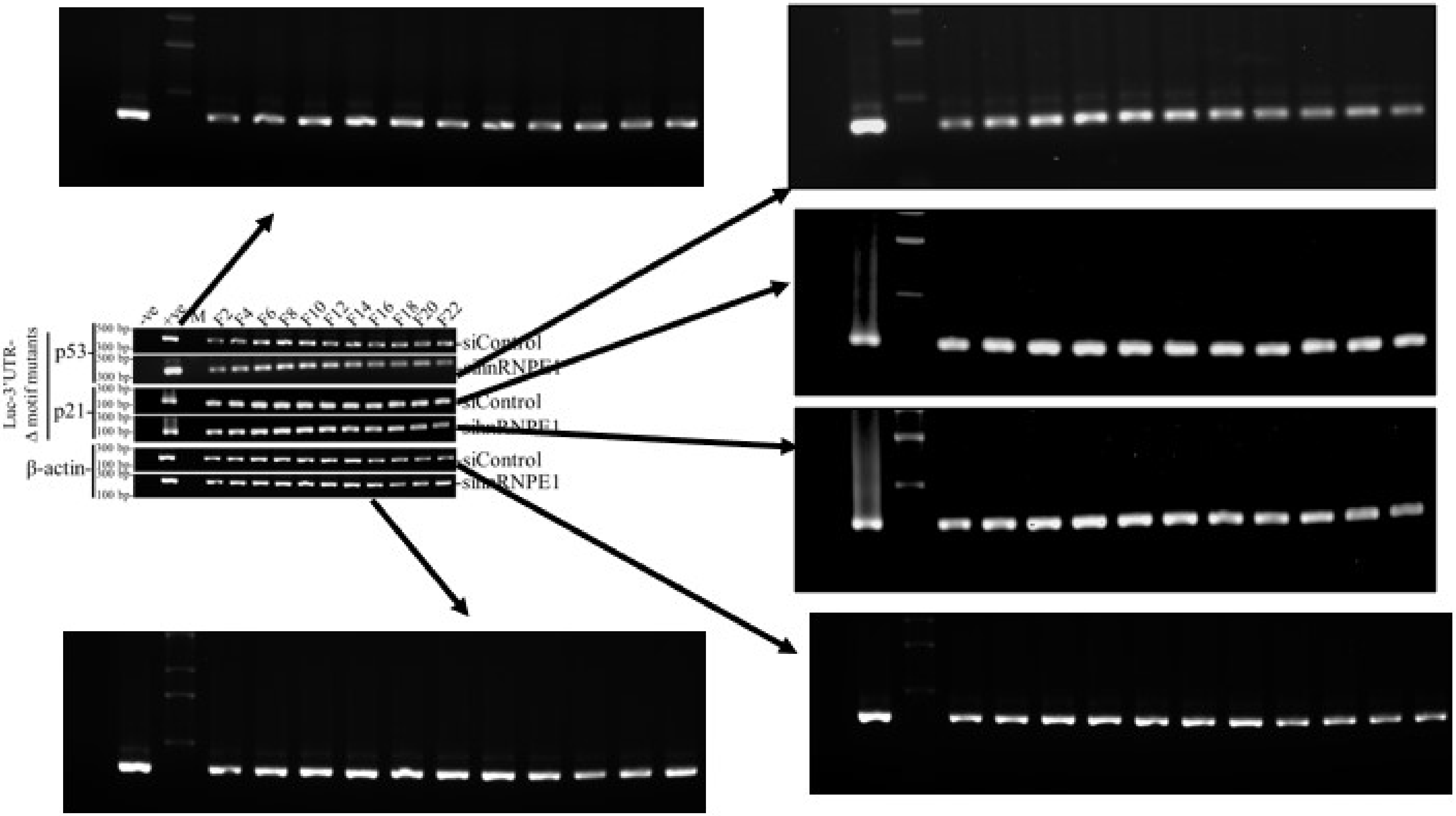

Supplement: Supplementary Figures [file mmc1.pdf]
