# Supplementary material for: The RNA-binding protein hnRNP E1 regulates p53 and p21 translation via KH1 and KH2 domain interactions with 3′ UTR C-rich motifs
Source: J Biol Chem. 2025 Dec 12;302(2):111042. doi: 10.1016/j.jbc.2025.111042 (PMC12816904; doi:10.1016/j.jbc.2025.111042)
Supplement: Supplementary Tables [file mmc2.docx]

**Supplementary table 1:** The details of the primers and sequences are presented in the table.

**Real-time primers:**

| **No.** | **Primer Name** | **Sequence (5’-3’)** |
| --- | --- | --- |
| 1. | E1-RT-F | TTCGGCTTCTTATGCACGGA |
| 2. | E1-RT-R | CTTACACCCGCCTTTCCCAA |
| 3. | P53-RT-F | CTCACCATCATCACACTGGAA |
| 4. | P53-RT-R | TCATTCAGCTCTCGGAACATC |
| 5. | P21-RT-F | ATGTCCGTCAGAACCCATGC |
| 6. | P21-RT-R | GCCATTAGCGCATCACAGTC |
| 7. | P16-RT-F | CCAACGCACCGAATAGTTACG |
| 8. | P16-RT-R | ACGGGTCGGGTGAGAGTG |
| 9. | KH1-RT-F | TCGGCTTCTTATGCACGGAA |
| 10. | KH1-RT-R | GGCCGGTCAGAGTGATGATT |
| 11. | KH2-RT-F | GATTGGGAAAGGCGGGTGTA |
| 12. | KH2-RT-R | CTGCTTGACACACTCGGTGA |
| 13. | KH3-RT-F | ACCCATGAACTCACCATTCCAA |
| 14. | KH3-RT-R | AACCTGCCTACCAGAGGAGC |
| 15. | Beta-Actin-F | CCTCGCCTTTGCCGATCC |
| 16. | Beta-Actin-R | TCGTCGCCCACATAGGAATC |
| 17. | 18S-F | GTAACCCGTTGAACCCCATT |
| 18. | 18S-R | CCATCCAATCGGTAGTAGCG |
| 19. | GAPDH-F | GGTATCGTGGAAGGACTCATGAC |
| 20. | GAPDH-R | ATGCCAGTGAGCTTCCCGTTCAGC |
| 21. | Fluc-RT-F | TCGTCACATCTCATCTACCT |
| 22. | Fluc-RT-R | TGATGGAATGGAACAACACT |
| 23. | hRluc-RT-F | CTGATCGGAATGGGTAAGTC |
| 24. | hRluc-RT-R | GATGGCCTTGATCTTGTCTT |

**Cloning primers:**

| **No.** | **Primer Name** | **Sequence (5’-3’)** |
| --- | --- | --- |
| 1. | E1-CF | GGTGGAATTCGCCATGGATGCCGGTGTGACTG |
| 2. | E1-CR | TAGACTCGAGGCTGCACCCCATGCCCTTCTCAG |
| 3. | P21 5’ UTR-CF | GAGAAAGCTTGAGGTGTGAGCAGCTGCCGA |
| 4. | P21 5’ UTR-CR | GCTAAAGCTTGGCGCCTCCTCCTCTGAGTGCCT |
| 5. | P21 3’ UTR-CF | GAGATCTAGATCCGCCCACAGGAAGCCTGCA |
| 6. | P21 3’ UTR-CR | GCTATCTAGAGGGCCCTAAAGTCACTAAGAATCA |
| 7. | KH1-CF | ATCATCGACTAGGAATTCTGCAGTCGACG |
| 8. | KH1-CR | GAATTCCTAGTCGATCATAGCGAAAGC |
| 9. | KH2-CF | ATTTGCCTGTAGGAATTCTGCAGTCGACG |
| 10. | KH2-CR | GAATTCCTACAGGCAAATCTGCTTGACAC |
| 11. | KH3-CF | AGTGGACTATTGGATGCATCTACTCAAAC |
| 12. | KH3-CR | TGCATCCAATAGTCCACTTTCAGTCACAC |
| 13. | P21-CF | CTCGGATCCATGTCAGAACCGGCTGGG |
| 14. | P21-CR | GTGGATCCTTAGTGATGGTGATGGTGATGTCCGGGCTTCCTCTTGGAGAAGATC |
| 15. | GST-E1-CF | TCCGAATTCATGGATGCCGGTGTGACTGA |
| 16. | GST-E1-CR | GCTCGAATTCCTAGCTGCACCCCATGC |
| 17. | GST-KH1-CF | TATGAATTCAATGTGACTCTCACCATTCG |
| 18. | GST-KH1-CR | TATGAATTCGATCATAGCGAAAGCCTTAA |
| 19. | GST-KH2-CF | TATGGATCCAGGCTGGTGGTGCCGGC |
| 20. | GST-KH2-CR | TATGGATCCAATCTGCTTGACACACTCGG |
| 21. | GST-KH3-CF | TATGGATCCACCACCCATGAACTCACC |
| 22. | GST-KH3-CR | TATGGATCCGATTAGATACTGGGCCAGAC |

**Deletion Primers:**

| **No.** | **Primer Name** | **Sequence (5’-3’)** |
| --- | --- | --- |
| 1. | E1-∆KH1-F | AGTGGACTAATCGACAAGCTGGAGGAAGA |
| 2. | E1-∆KH1-R | CTTGTCGATTAGTCCACTTTCAGTCACAC |
| 3. | E1-∆KH2-CF | GTCACCCTGTGCCTGGTCATGCTGGAGAC |
| 4. | E1-∆KH2-R | TGACCAGGCACAGGGTGACCGGGGGC |
| 5. | E1-∆KH3-F | TCTACTCAAAATGCCAGGCTTTCCTCTGA |
| 6. | E1-∆KH3-R | CCTGGCATTTTGAGTAGATGCATCCAAACTTG |
| 7. | P53-3’UTR-∆M1-F | AACCCAAAAGGGTGGGAGGCTGTGAGTG |
| 8. | P53-3’UTR-∆M1-R | CTCCCACCCTTTTGGGTTTTGGGTCTTTG |
| 9. | P53-3’UTR-∆M2-F | GACCCAGTCGACCCTGAGCATAAAACAAG |
| 10. | P53-3’UTR-∆M2-R | CTCAGGGTCGACTGGGTCTCGCTTTGTTG |
| 11. | P53-3’UTR-∆M3/4-F | AAAAGGGAGTGCAGATGTGCTTGCAGAA |
| 12. | P53-3’UTR-∆M3/4-R | ACATCTGCACTCCCTTTTTATATCCCAT |
| 13. | P21-3’UTR-∆M1-F | AATTCCCCTTCCCTTCAGTACCCTCTCAG |
| 14. | P21-3’UTR-∆M1-R | CTGAAGGGAAGGGGAATTGCAGAGCCCAG |
| 15. | P21-3’UTR-∆M2-F | GAGGTGCCTGACTGGAAGGGGAAGGGACA |
| 16. | P21-3’UTR-∆M2-R | CTTCCAGTCAGGCACCTCAGAGCCACCTG |
| 17. | P21-3’UTR-∆M3-F | AGCGACCTTCACTTTGATTAGCAGCGGAA |
| 18. | P21-3’UTR-∆M3-R | ATCAAAGTGAAGGTCGCTGGACGATTTGA |

**Universal primers:**

| **No.** | **Primer Name** | **Sequence (5’-3’)** |
| --- | --- | --- |
| 1. | CMV-F | CGCAAATGGGCGGTAGGCGTG |
| 2. | BGH-R | TAGAAGGCACAGTCGAGG |
| 3. | T7-HSVTK-F | ATTAATACGACTCACTATAGGCATATTAAGGTGACGCGTGTG |
| 4. | T7-hRluc-F | ATTAATACGACTCACTATAGGACCATGGCTTCCAAGGTGTAC |
| 5. | T7-Fluc-F | ATTAATACGACTCACTATAGGCATGGAAGACGCCAAAAACAT |
| 6. | T7-SV40-F | ATTAATACGACTCACTATAGGTTTGGAGGCCTAGGCTTTTG |
| 7. | Fluc-F | ACCATGGCTTCCAAGGTGTAC |
| 8. | LacZ-R | GACAGTATCGGCCTCAGGAA |
| 9. | Rluc-F | CCAGGATTCTTTTCCAATGC |
| 10. | Rluc-R | AGAATTACTGCTCGTTCTTCAG |
| 11. | SV40polyA-R | TTGTGGTTTGTCCAAACTCA |
| 12. | F1ori-F | GTGGACTCTTGTTCCAAACTGG |
| 13. | F1ori-R | AGGGAAGAAAGCGAAAGGAG |
| 14. | LucNrev | CCTTATGCAGTTGCTCTCC |
| 15. | Amp-R | ATAATACCGCGCCACATAGC |
| 16. | T7 | TAATACGACTCACTATAGGG |
| 17. | EBV-rev | GTGGTTTGTCCAAACTCATC |
| 18. | T7-p53-3’UTR-F | ATTAATACGACTCACTATAGGACATTCTCCACTTCTTGTTCCC |
| 19. | T7-p21-3’UTR-F | ATTAATACGACTCACTATAGGATCCGCCCACAGGAAGCC |
